# A Dual‐Mode In Situ EV‐miRNA Profiling Platform for Machine‐Learning‐Assisted Gastric Cancer Liquid Biopsy

**DOI:** 10.1002/advs.77483

**Published:** 2026-09-06

**Authors:** Wenbin Li, Rui Fan, Kun Xie, Jiehua Zhong, Yitong Zhu, Tingting Ji, Yuanyuan Qin, Shijin Peng, Yu Zhang, Yuhang Guo, Tiange Zhang, Chunchen Liu, Bo Li, Lei Zheng, Ye Zhang, Xiaohui Yan

**Affiliations:** ^1^ Department of Laboratory Medicine Nanfang Hospital Southern Medical University Guangzhou People's Republic of China; ^2^ Guangdong Provincial Key Laboratory of Proteomics, Medical School of Basic Medical Sciences Research Center of Nanfang Hospital, Nanfang Hospital Southern Medical University Guangzhou People's Republic of China; ^3^ Guangdong Provincial Key Laboratory of Precision Medical Diagnostics Guangdong Engineering and Technology Research Center for Rapid Diagnostic Biosensors Guangdong Provincial Key Laboratory of Single‐cell and Extracellular Vesicles Guangdong Engineering Research Center of Advanced In Vitro Diagnostic Technology Nanfang Hospital Southern Medical University Guangzhou People's Republic of China; ^4^ Guangdong Provincial Clinical Research Center For Laboratory Medicine Guangzhou People's Republic of China; ^5^ State Key Laboratory of Multi‐Organ Injury Prevention and Treatment, Southern Medical University; National Clinical Research Center for Kidney and Urological Diseases, Nanfang Hospital Southern Medical University Guangzhou People's Republic of China

## Abstract

EV‐miRNAs are promising gastric cancer (GC) biomarkers for early diagnosis, yet their clinical application is limited by major detection challenges arising from the low abundance and high sequence homology of EV‐miRNAs. Herein, we developed a dual‐mode platform for sensitive EV‐miRNA in situ profiling based on liposome‐encapsulated localized exponential catalytic hairpin assembly (L‐LECHA). L‐LECHA enables the localized exponential catalytic hairpin assembly system to be delivered into EVs and activates exponential signal amplification, resulting in sensitive and rapid detection of EV‐miRNAs. Based on the L‐LECHA, this platform achieved a limit of detection of 52.48 aM, representing a 10.96‐fold improvement over typical ECHA. Compared to single‐mode detection, the dual‐mode platform demonstrated a wider linear detection range and greater accuracy. By combining an EV‐miRNA panel with a k‐nearest neighbors (KNN) model, this platform achieved an AUC of 0.974 for diagnosis of GC. It can also be used for pathological classification of GC. This work provides a robust tool for GC liquid biopsy and precision management.

## Graphical abstract

An in situ dual‐mode platform integrates fusogenic liposomes with localized autocatalytic DNA circuits for plasma‐derived extracellular vesicle microRNA analysis. Following membrane fusion, target miRNAs trigger feedback‐assisted LECHA amplification inside intact vesicles, generating fluorescence and electrochemical outputs. Machine learning integrates these complementary signals to enable sensitive discrimination of gastric cancer patients from healthy donors.

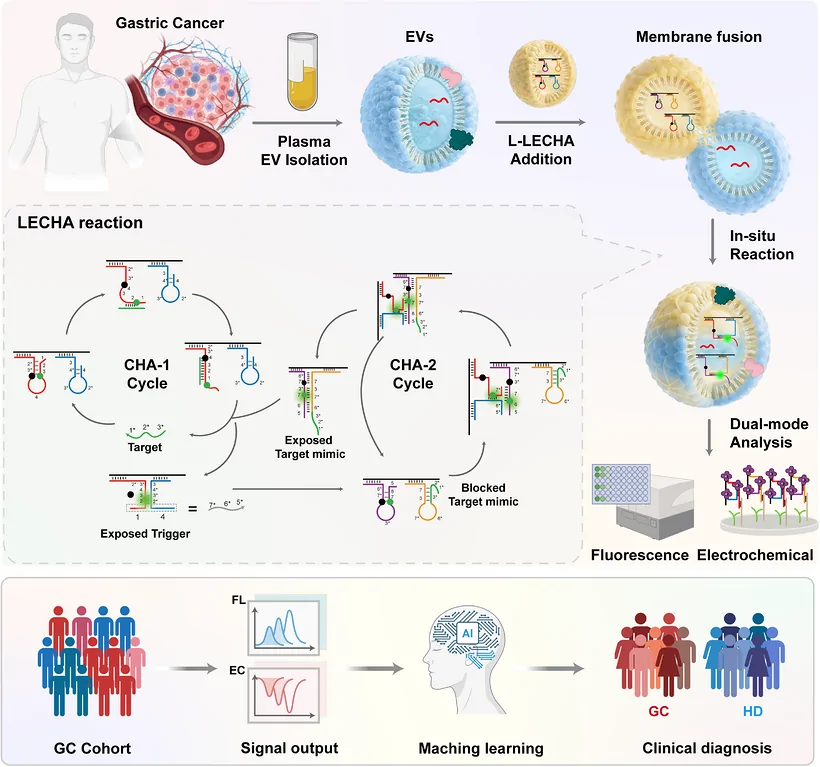

## 1 Introduction

GC is one of the most prevalent malignant tumors worldwide, ranking fourth in global cancer incidence and fourth in cancer‐related mortality [1, 2, 3, 4]. Current routine clinical strategies for GC management, including gastroscopy, tissue biopsy and cross‐sectional imaging, have notable limitations: gastroscopy and biopsy are invasive and operator‐dependent, while imaging modalities have insufficient sensitivity for early lesions and poor accuracy in differentiating pathological subtypes preoperatively [5, 6, 7, 8]. These limitations lead to delayed diagnosis, inappropriate treatment selection, and unfavorable prognosis for a large proportion of GC patients [9]. Therefore, developing noninvasive, sensitive, and reliable detection methods to obtain comprehensive tumor biological information is of great importance for optimizing the full‐cycle clinical management of GC [10, 11, 12]. EVs, which are key mediators of intercellular communication, deliver a variety of encapsulated bioactive molecules, including miRNAs, from parental cells to recipient cells, and are deeply involved in the entire process of GC tumorigenesis, invasion, metastasis, and malignant progression [13, 14, 15, 16, 17, 18]. Clinical evidence has confirmed that EV‐miRNAs exhibit distinct expression profiles in GC patients, with expression patterns closely correlated with tumor occurrence and development [5, 13, 19, 20, 21].

Currently, reverse transcription quantitative polymerase chain reaction (RT‐qPCR) is widely accepted as the gold standard for EV‐miRNA quantification, but it involves a complex workflow—including EV lysis, RNA extraction, reverse transcription and multi‐step amplification—that inevitably causes target miRNA degradation and sample loss, leading to poor detection reproducibility and large quantitative deviations between batches [19, 22, 23, 24, 25, 26, 27, 28]. In situ detection enables direct quantification of target miRNAs in individual intact EVs without EV lysis and RNA extraction, which maximally preserves sample integrity, avoids target loss and degradation, simplifies the operation workflow, and retains information on EV heterogeneity [29, 30]. However, conventional in situ detection tools, such as molecular beacons and nanoflares, produce only a 1:1 target‐to‐signal output [31, 32, 33]. The nanoscale volume of individual EVs leads to extremely low copy numbers of target miRNAs per vesicle, resulting in ultra‐weak signal output and insufficient detection sensitivity for low‐abundance GC‐related biomarkers [34]. This insensitivity leads to the loss of weak biological signals associated with GC diagnosis and pathological characteristics, greatly restricting the comprehensive clinical application of in situ EV‐miRNA detection [35, 36].

To address this challenge, a variety of in situ signal amplification strategies have been developed, mainly categorized into enzyme‐mediated amplification systems and enzyme‐free DNA amplification circuits. Enzyme‐mediated systems have high amplification efficiency, but are limited by strict reaction conditions, compromised enzyme activity in the complex intravesicular environment, and poor stability in multi‐marker panel detection [37, 38, 39, 40]. Traditional enzyme‐free linear amplification circuits, represented by catalytic hairpin assembly (CHA), have excellent biocompatibility and stability, but suffer from moderate amplification efficiency and slow reaction kinetics, failing to meet the demand for ultra‐sensitive and rapid detection of low‐abundance EV‐miRNAs [41, 42, 43, 44, 45]. Exponential catalytic hairpin assembly (ECHA) has attracted increasing attention in recent years: through a target‐triggered positive feedback loop, the generated amplification products can in turn initiate the circuit, realizing enzyme‐free exponential signal amplification with significantly higher signal gain and faster reaction kinetics than traditional CHA [46, 47, 48]. However, the inherent, non‐negligible background leakage cannot be avoided due to thermodynamically driven non‐specific probe hybridization. Although modification of the CHA structure can decrease background leakage, reaction kinetics and signal gain are affected. Thus, in ECHA, the balance between reaction kinetics and background leakage remains challenging. In addition, most existing in situ amplification strategies rely on single‐mode signal output, which is susceptible to interference from complex clinical biofluid matrices, leading to unstable quantitative results of multi‐marker panels in different clinical samples [49, 50, 51]. Therefore, there is still a lack of an in situ EV‐miRNA detection platform that can achieve high sensitivity and accuracy, which hinders the clinical application of EV‐miRNA biomarkers in GC management.

**SCHEME 1:**
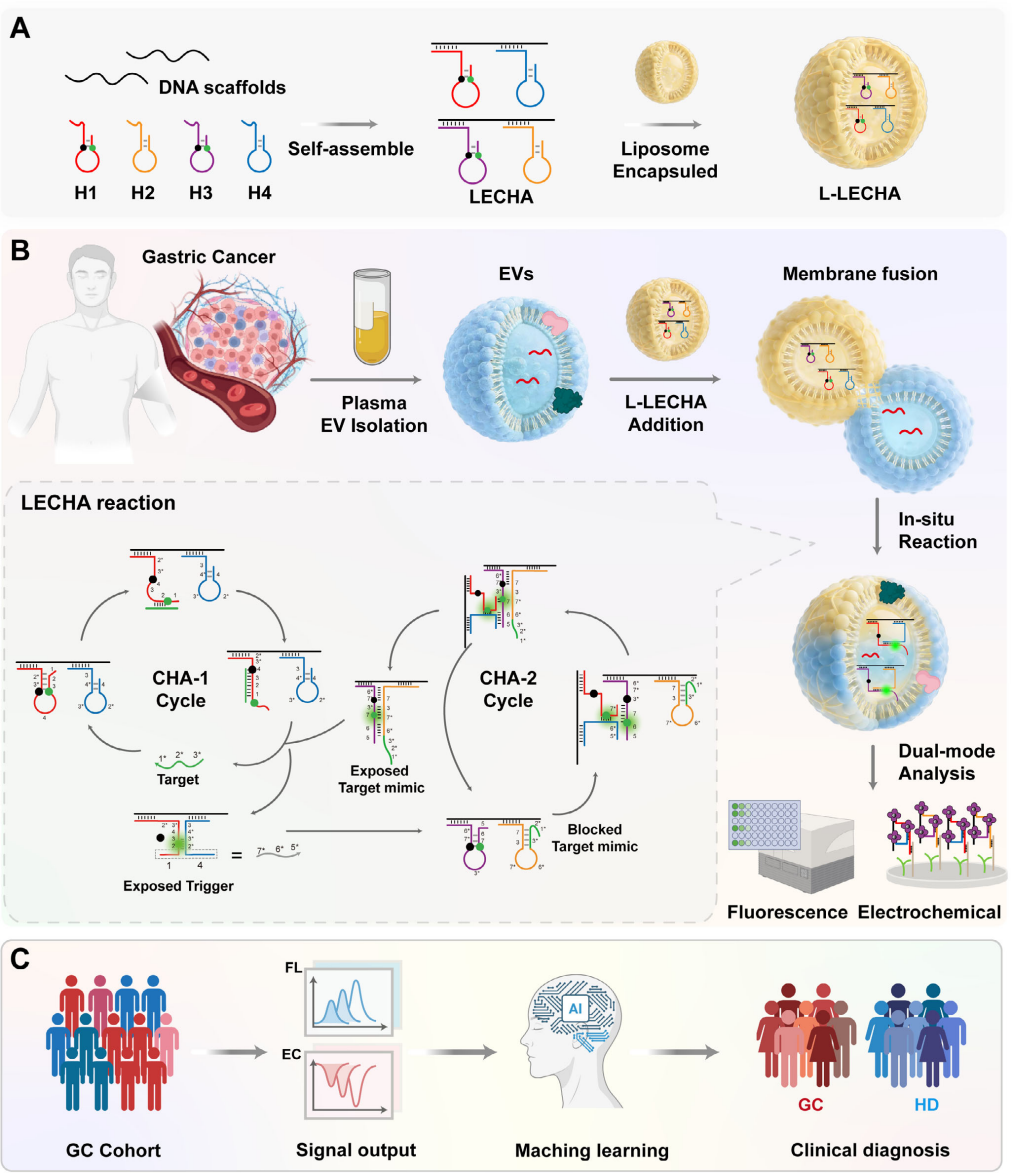
Schematic illustration of the L‐LECHA analysis platform. (A) The construction of L‐LECHA. (B) The reaction principle of the L‐LECHA. (C) The clinical workflow of the L‐LECHA analysis.

Herein, we developed an in situ dual‐mode platform for sensitive EV‐miRNA profiling based on liposome‐encapsulated localized exponential catalytic hairpin assembly (L‐LECHA), which enables the localized exponential catalytic hairpin assembly system to be delivered into EVs and generates a strong fluorescence signal, resulting in sensitive and rapid detection of EV‐miRNAs. Specifically, the LECHA probe was self‐assembled from four CHA hairpins and a DNA scaffold. Subsequently, the LECHA was encapsulated in a liposome to form L‐LECHA (Scheme 1A). During detection, L‐LECHA delivers the LECHA circuit to EVs through a fusion‐compatible membrane interaction. The LECHA was then activated by the input EV‐miRNA. Due to the proper design of the DNA scaffold and CHA hairpins, background leakage was restrained through thermodynamic optimization. Moreover, reaction kinetics and signal gain also benefited from the localized effect of CHA substrates, generating a strong and rapid signal output. The intravesicular reaction was measured directly in fluorescence mode, whereas electrochemical readout was performed after the in situ reaction by lysing and measuring the released products by DPV. These complementary readouts extend the measurable concentration range and improve the robustness of marker integration. Using GC‐associated EV‐miR‐1246 as a model target, we verified the excellent analytical performance of the L‐LECHA platform, with a limit of detection of 52.48 aM. When further coupled with a GC‐specific EV‐miRNA panel and machine learning model, the L‐LECHA platform realized accurate non‐invasive diagnosis of GC (AUC = 0.974) and pathological subtype prediction (accuracy of 87%) in clinical cohorts. We anticipate that this dual‐mode L‐LECHA platform will provide a robust and versatile tool for tumor‐derived EV‐miRNA analysis and hold great promise for advancing comprehensive GC clinical management.

## 2 Results

### 2.1 Feasibility of Dual‐Mode Analysis Using Localized Exponential Catalytic Hairpin Assembly (LECHA)

**FIGURE 1:**
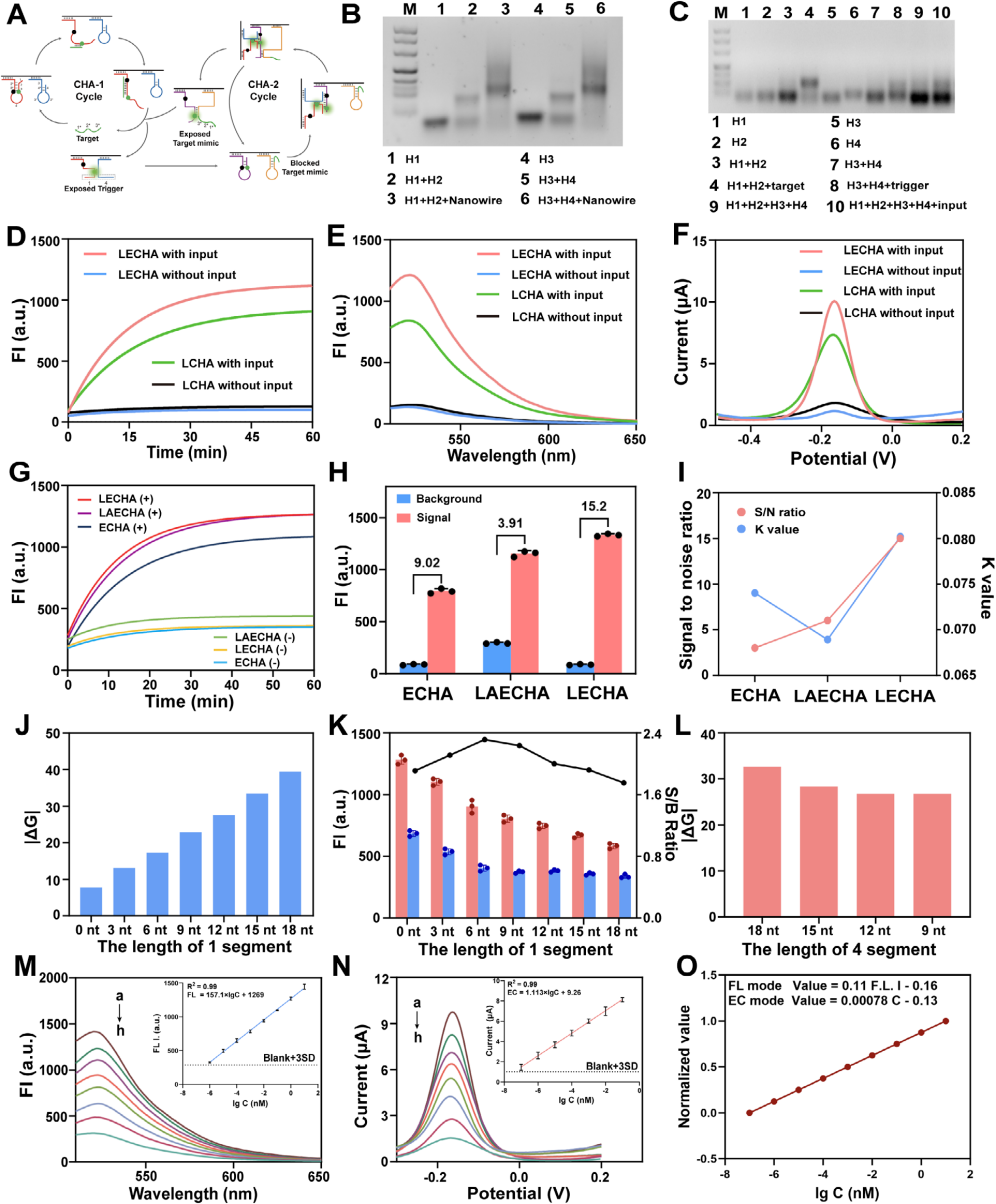
The feasibility of the dual‐mode analysis via LECHA. (A) The scheme of the LECHA reaction. (B) 2% agarose gel electrophoresis analysis of LECHA construction. (C) Agarose gel electrophoresis confirmation of LECHA activation, including the individual scaffold‐localized CHA products and reciprocal product‐mimic controls; lane identities and input species are specified in the gel annotation. (D) Fluorescence kinetic monitoring of the LECHA reaction and LCHA (input 10 nM). (E,F) The fluorescence and electrochemical feasibility analysis of the proposed LECHA and LCHA (input 10 nM, 30 min). (G) Fluorescence kinetic monitoring of the LECHA, LAECHA, and ECHA reaction (input 10 nM, 30 min). (H) Signal and background analysis of the LECHA, LAECHA and ECHA reaction. (I) Signal‐to‐noise ratio and kinetic K‐value analyses of the LECHA, LAECHA, and ECHA reaction. (J) NUPACK free‐energy analysis of candidate segment 1/segment 4 designs in H2. (K) Fluorescence signal and background leakage of LECHA with different segment 1/segment 4 designs. (L) NUPACK free‐energy analysis of the corresponding segment 1/segment 4 designs in H3. (M) The fluorescence responses of LECHA and the corresponding calibration curve over an input RNA concentration range of 10 fM to 10 nM. (N) The electrochemical responses of LECHA and the corresponding calibration curve over an input RNA concentration range of 100 aM to 100 pM. (O) The correction equation of the dual‐mode analysis. All error bars are the standard deviations by three repetitive assays (*n* = 3).

The LECHA was constructed by employing two 10‐nt DNA scaffolds to link the substrates of two CHA systems, enabling the two systems to undergo autocatalytic feedback‐assisted, exponential‐like amplification. Specifically, CHA‐1 can be activated by the input strand and the CHA‐2 product, while CHA‐2 can be activated by the CHA‐1 product (Figure 1A). With this design, the distance between the hairpin substrates of each CHA system was significantly reduced, thereby generating a localized effect. Through analysis using the collision equation, the localized concentration was calculated to be 4.6 × 10^5^ nM, which was 4600‐fold higher than that of the free‐collision CHA substrate (Figure S1). This design provided several advantages: once the input strand was present, the CHA‐1 product can be rapidly generated and can continuously drive the CHA‐2 reaction. In turn, the CHA‐2 product accumulated an input‐like domain that accelerated CHA‐1, forming an autocatalytic feedback cycle that regenerates trigger‐like species and supports repeated amplification. To confirm the construction of the LECHA, agarose gel electrophoresis was employed. As illustrated in Figure 1B, the gradual addition of CHA hairpins showed almost the same molecular weight (Lanes 1–2 and 4–5). After the addition of the DNA scaffold, a higher‐molecular‐weight band appeared (Lanes 3 and 6), indicating the successful assembly of the LECHA. To validate the reaction feasibility of LECHA, gel electrophoresis was first performed. The gel results confirmed successful triggering of LECHA by the input miR‐1246 strand. Lane 8 contained a synthetic trigger mimic corresponding to the input‐like sequence exposed by the upstream CHA product, whereas lane 10 contained the synthetic miR‐1246 target mimic. Additional product‐mimic controls showed that the CHA‐1 product activated CHA‐2 and that the CHA‐2 product activated CHA‐1, supporting reciprocal activation of the two localized modules (Figure 1C). Then, the reaction kinetics of LECHA were monitored. As illustrated in Figure 1D, the LECHA reached the signal peak within 30 min, representing a 1.3‐fold reduction in reaction time compared with the localized cascade CHA (LCHA) (Figure S2). This feedback‐assisted architecture also provided a moderate signal gain, which was 1.24‐fold higher than that of the LCHA. Fluorescence spectral analysis and differential pulse voltammetry (DPV) also confirmed the signal gain (1.46‐fold in fluorescence and 1.37‐fold in DPV) (Figure 1E,F and Figure S3). Together with the product‐mimic controls, these moderate kinetic and signal improvements support an autocatalytic feedback‐assisted, exponential‐like amplification pathway (Figure S4).

To demonstrate the superiority of LECHA, we employed exponential CHA without scaffold localization (ECHA) and scaffold‐localized all‐exponential CHA (LAECHA) for analysis. As shown by the reaction kinetics in Figure 1G, the LECHA and LAECHA exhibited similar reaction kinetics when triggered by input miR‐1246, but LAECHA showed significant background signal leakage. Although ECHA showed almost no background signal leakage, its signal output and reaction kinetics were reduced. Further analysis of signal output and background leakage using a fluorescence spectrometer showed that LECHA achieved a signal‐to‐noise ratio of 15.2, which was significantly higher than those of ECHA and LAECHA (Figure 1H). This may be due to the appropriate localization of exponential amplification, whereas excessive localization increases the background leakage (Figures S5 and S6). The kinetic parameters also confirmed the best balance between reaction kinetics and background leakage in LECHA (Figure 1I). Moreover, we studied the substrate distances in LECHA (5, 10, 15 and 20 nt), which produced different localization effects for the CHA substrates; the 20‐nt distance produced a low local concentration, whereas the 5‐nt distance produced a high local concentration. As shown in Figure S7, LECHA localized by the 20‐nt scaffold exhibited slow reaction kinetics. Although the 5‐nt scaffold produced the strongest signal output, background signal leakage increased. Only LECHA localized at a 10‐bp distance balanced the kinetics and background leakage, indicating the need for an appropriate localization design. The trigger design in CHA‐1 was important for reaction kinetics (Segment 1 and segment 4), which together form the trigger sequence that activates CHA‐2. NUPACK was used to evaluate the thermodynamic stability of the candidate hairpin structures. Increasing the length of segment 1 increased structural stability, but excessive stabilization reduced trigger accessibility and LECHA signal output (Figure 1J–L). Because the total trigger length was fixed, the lengths of segments 1 and 4 were varied in a coordinated manner. A 6‐nt segment 1 provided the best balance between target‐triggered signal, background leakage, and signal‐to‐noise ratio.

The advantages of the LECHA encouraged us to study the analytical performance after optimization of the key conditions (reaction time: 30 min, reaction temperature: 25°C) (Figure S8). First, the sensitivity was explored by detecting serially diluted input miR‐1246. As illustrated in Figure 1M,N, FL and DPV signals were positively correlated with the input miR‐1246 concentration, with linear regression fitting equations of FL = 157.1 logC + 1269 and I = 1.113 logC + 9.261, respectively. Moreover, according to the background + 3SD rule, the limit of detection (LOD) was calculated as 549.54 aM (FL mode) and 52.48 aM (EC mode). Interestingly, the FL mode provided a strong response over the higher‐concentration range (1 fM to 10 nM), whereas the EC mode retained greater sensitivity at lower input concentrations (100 aM to 100 pM). Thus, the two modes contribute complementary analytical information rather than simply duplicating the same measurement: fluorescence provides robust signal output at higher concentrations, while electrochemical detection improves sensitivity at low abundance. By constructing a correction equation, the dual‐mode detection of input miR‐1246 achieved a detection range from 100 aM to 10 nM (Figure 1O).

### 2.2 Construction of the In Situ EV‐miRNA Analysis Platform Based on Liposome‐Encapsulated LECHA (L‐LECHA)

**FIGURE 2:**
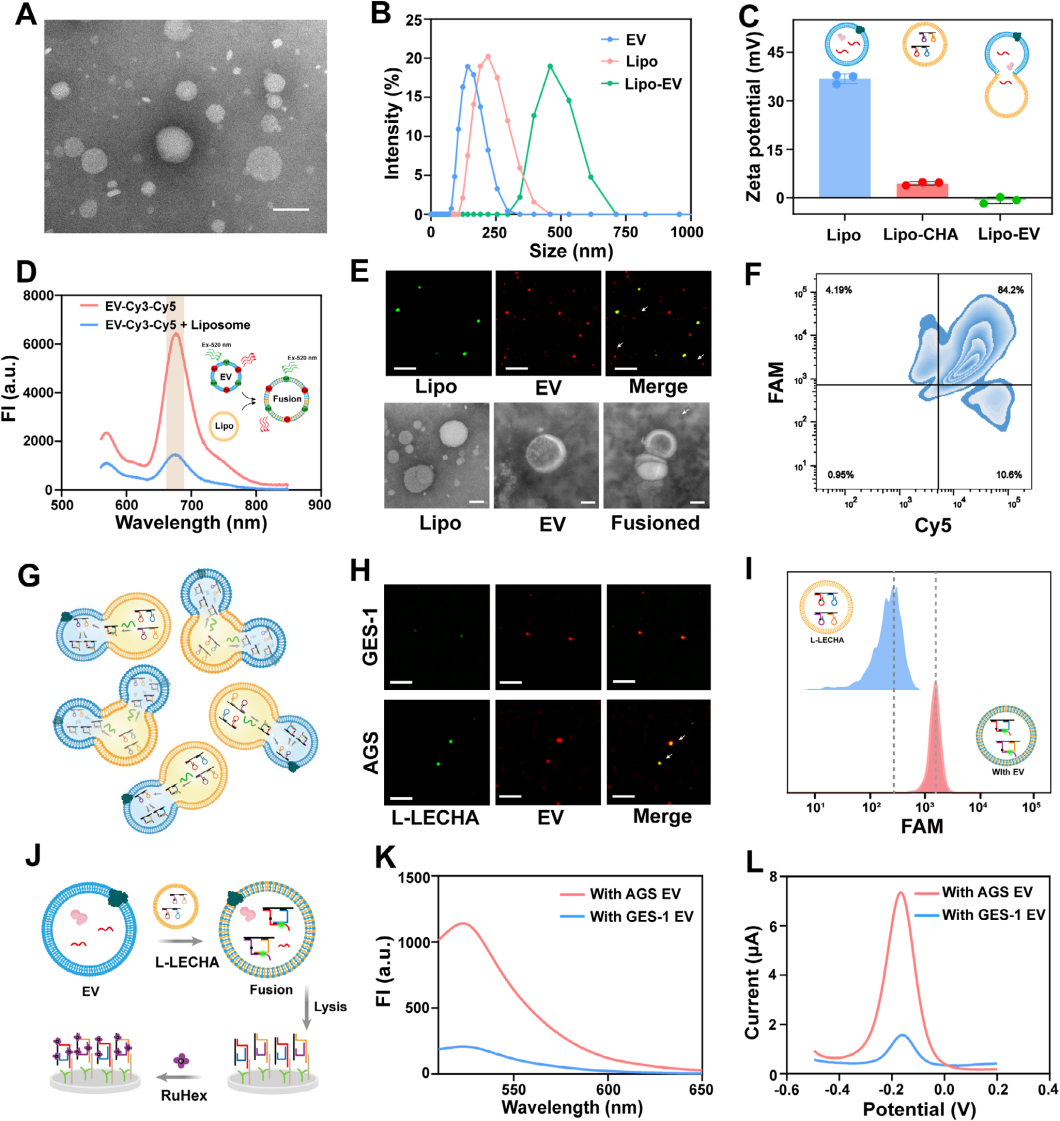
Construction of the in situ EV‐miRNA analysis platform based on LECHA. (A) The TEM image of the proposed L‐LECHA. (B) DLS analysis of L‐LECHA, EVs, and the mixed vesicle population; the size shift is interpreted as vesicle association or complex formation (EV: 1.01 × 10^9^ Particles/mL, L‐LECHA: 2.47 × 10^9^ particles/mL). (C) The Zeta potential analysis of the fusion of the L‐LECHA and EVs. (D) The FRET analysis of the fusion between the L‐LECHA and EVs (30 min). (E) Super‐resolution microscopy and TEM characterization of the interaction between EVs and L‐LECHA (30 min). (F) Representative NanoFCM analysis of CD63‐positive EVs and liposome‐derived fluorescent cargo after incubation. (G) Schematic of L‐LECHA delivery illustrating that one‐to‐one and one‐to‐many vesicle interactions may occur. (H) The super‐resolution optical microscopy images of the EV‐miR‐1246 analysis in GES‐1 EVs and AGS EVs using L‐LECHA. (I) The Nanoflow cytometry analysis of the expression of EV‐miR‐1246 in AGS EVs using L‐LECHA. (J) Workflow for the direct fluorescence readout of the intravesicular reaction and post‐reaction electrochem*ical* detection using a parallel aliquot. (K) The fluorescence spectroscopy analysis of the in situ analysis of EV using L‐LECHA (30 min). (L) The DPV analysis of the in situ analysis of EV using L‐LECHA (30 min). All error bars are the standard deviations by three repetitive assays (*n* = 3).

Cationic liposomes were used to encapsulate LECHA and facilitate close contact with the negatively charged EV membrane. L‐LECHA was prepared by rotary evaporation and displayed a uniform morphology with a diameter of approximately 250 nm (Figure 2A), with approximately 55 LECHA molecules per L‐LECHA particle according to the calculation (Figure S9). EVs isolated from AGS cells had an average diameter of approximately 100 nm and high purity (Figure S10). DLS, zeta‐potential, and UV–Vis measurements further demonstrated changes in particle size and surface charge after incubation, indicating the interaction between EV and L‐LECHA (Figure 2B,C and Figure S11). Then, membrane lipid mixing between L‐LECHA and EVs was evaluated using a Cy3/Cy5‐CHOL FRET assay. Cy3‐CHOL and Cy5‐CHOL were co‐inserted into the same EV membrane, where their close proximity generated efficient FRET before incubation with unlabeled L‐LECHA. Following incubation, sensitized Cy5 emission decreased markedly, indicating dilution and increased spatial separation of the membrane‐anchored fluorophores as a result of membrane lipid redistribution (Figure 2D). This result supports lipid mixing between the EV and liposome membranes. Moreover, PKH67‐labeled L‐LECHA and PKH26‐labeled EVs showed extensive fluorescence colocalization, indicating lipid mixing between EV and L‐LECHA (Figure 2E). Because PKH dyes can undergo membrane redistribution, an orthogonal labeling strategy was subsequently employed: EVs were identified using a Cy5‐conjugated anti‐CD63 antibody, whereas the liposomes contained FAM‐labeled DNA probes. Super‐resolution microscopy and NanoFCM showed increased CD63/FAM double‐positive particles following incubation, supporting the association of liposome‐derived cargo with EVs. Finally, comparison of liposomes with different surface charges showed that cationic DOTMA liposomes produced markedly greater EV interaction and cargo‐positive events than neutral DOPC and anionic DPPG liposomes under identical conditions (Figures S12 and S13). Collectively, these results support the role of electrostatic attraction in promoting close membrane contact and facilitating the subsequent fusion process.

To study the analytical feasibility of L‐LECHA, EVs were isolated from AGS cells and GES‐1 cells, in which miR‐1246 is differentially expressed. Following the mixed L‐LECHA with EVs, the delivered LECHA probes responded to intravesicular miR‐1246 (Figure 2G). Super‐resolution fluorescence microscopy showed stronger signal output from AGS EVs than from GES‐1 EVs, and NanoFCM showed the same expression trend (Figure 2H,I). AGS EVs produced higher fluorescence and DPV signals than GES‐1 EVs (1170 a.u. and 7.346 µA, respectively), representing 5.44‐fold and 4.74‐fold increases (Figure 2J–L). Accordingly, L‐LECHA is described as enabling an in situ intravesicular reaction with direct fluorescence readout and post‐reaction electrochemical detection.

We next compared an in situ intravesicular reaction with a workflow in which EVs were lysed before LECHA amplification. In FBS‐treated samples, the pre‐lysis workflow generated a weaker signal than the intravesicular workflow (Figure S14). Moreover, the pre‐lysis workflow remained slightly lower even without FBS. These results demonstrated the advantages of in situ reaction of L‐LECHA, which shown a higher signal output.

### 2.3 RNA Analysis in Cell Line EV Using Dual‐Mode L‐LECHA

**FIGURE 3:**
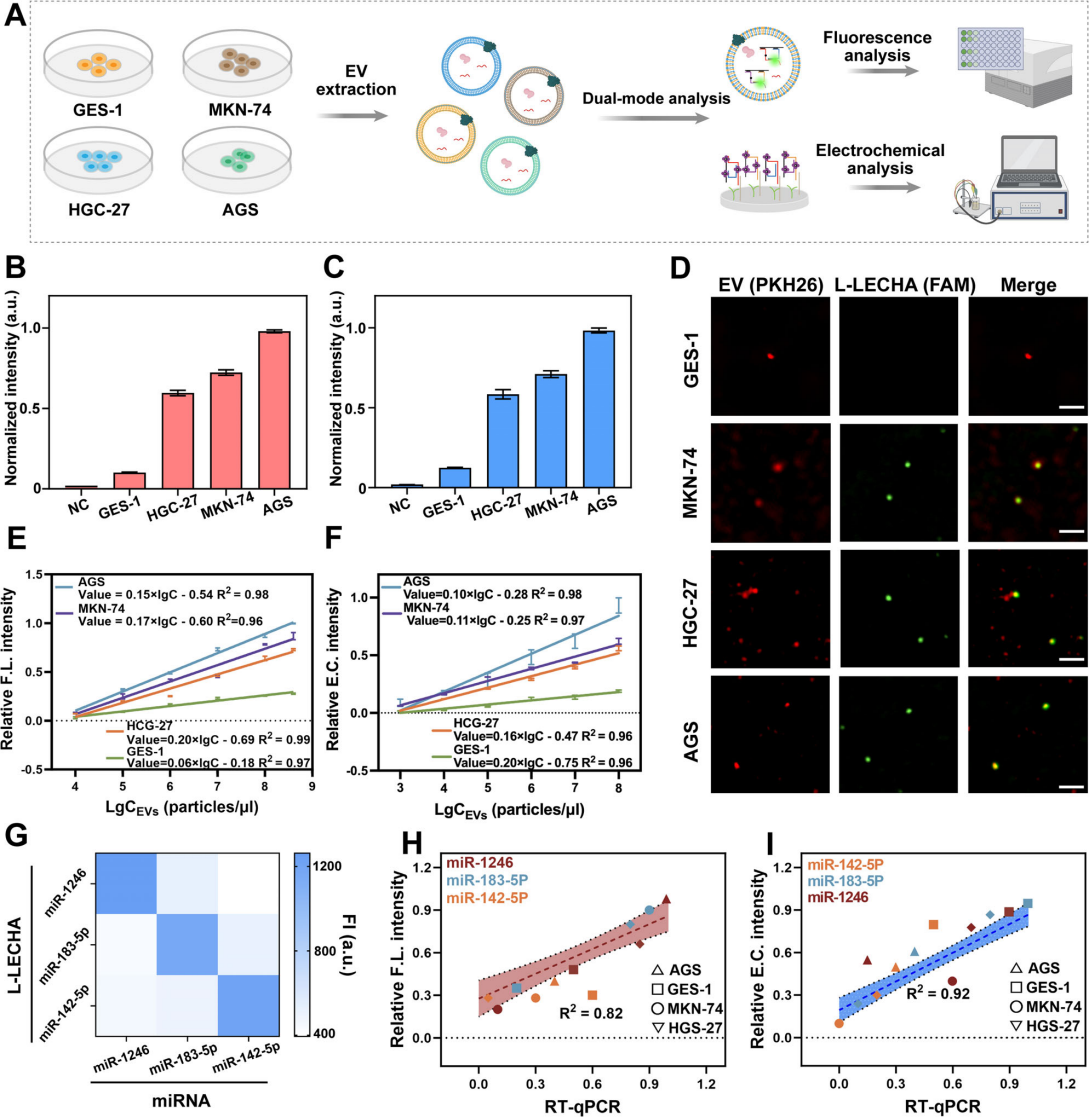
RNA analysis in cell line EV using dual‐mode L‐LECHA. (A) Schematic illustration of the in situ analysis of the cell line EV using dual‐mode L‐LECHA. (B) The in situ EV‐miRNA‐1246 fluorescence analysis of the GES‐1, MKN‐74, HGC‐27, and AGS cell line EV using L‐LECHA. (C) The in situ EV‐miRNA‐1246 DPV analysis of the GES‐1, MKN‐74, HGC‐27, and AGS cell line EV using L‐LECHA. (D) The super‐resolution optical microscopy images of in situ EV‐miRNA‐1246 analysis in GES‐1, MKN‐74, HGC‐27, and AGS cell line EVs using L‐LECHA. (E) The fluorescence response of serially diluted cell‐line EVs, including GES‐1, MKN‐74, HGC‐27, and AGS, using L‐LECHA (30 min). (F) The DPV signal response of serially diluted cell‐line EVs including GES‐1, MKN‐74, HGC‐27, and AGS using L‐LECHA (30 min). (G) Heat map analysis of the in situ orthogonal identification of three sEV‐miRNAs in fluorescence mode (30 min). (H,I) The correlation between L‐LECHA and RT‐qPCR methods for in situ dual‐mode detection of the expression of three sEV‐miRNAs in sEVs derived from GES‐1, MKN‐74, HGC‐27, and AGS cell lines. The bright red dashed line indicates the best linear fit in the log–log scale, and the shaded red area represents the 95% confidence interval. All error bars are the standard deviations by three repetitive assays (*n* = 3).

To test the proposed L‐LECHA in an example application, we applied L‐LECHA for dual‐mode RNA analysis of EV derived from three gastric cancer cell lines (AGS, MKN‐74 and HGC‐27) and a gastric mucosal epithelial cell line (GES‐1) (Figure 3A). In situ fluorescence signal analysis of miR‐1246 showed that the fluorescence intensities of tumor‐cell EVs were highest for AGS, followed by MKN‐74 and HGC‐27. All of these intensities were significantly higher than those of EVs from the GES‐1 cell line (Figure 3B). The electrochemical signals showed a normalized pattern similar to the fluorescence output (Figure 3C). In addition, the super‐resolution microscopy results were also consistent with the expression pattern of miR‐1246 among the cell lines (Figure 3D). These results indicate the high specificity of the proposed L‐LECHA. As shown in Figure 3E,F, strong linear correlations were observed between the fluorescence and electrochemical signals and the EV concentration for each cell line, with correlation coefficients (R^2^) above 0.96, highlighting the high accuracy and reliability of the L‐LECHA for EV‐miRNA analysis.

To select the EV‐miRNA panel, we analyzed EV‐miRNA sequencing data from the GEO dataset GSE332730. Differential‐expression analysis between the GC and HD groups identified 49 upregulated EV‐miRNAs using the predefined criteria *p* < 0.05 and fold change > 3. Heatmap analysis showed distinct expression patterns between the two groups (Figure S15). We then cross‐referenced these candidates with EV‐miRNAs previously associated with gastric cancer in the literature [5]. The overlap between the sequencing‐derived candidates and literature‐supported markers yielded EV‐miR‐1246, EV‐miR‐183‐5p, and EV‐miR‐142‐5p, which were selected for the final panel. After confirming assay specificity by orthogonal testing, we profiled the three EV‐miRNAs in EVs derived from the four cell lines (Figure 3G and Figure S16). Most signals were higher in gastric‐tumor‐cell EVs than in GES‐1 EVs, particularly for EV‐miR‐1246 and EV‐miR‐183‐5p (Figure 3H,I). RT‐qPCR showed expression trends consistent with the dual‐mode L‐LECHA measurements (R^2^ = 0.82 for fluorescence and R^2^ = 0.95 for electrochemical detection), supporting the analytical applicability of the platform.

### 2.4 Clinical Feasibility of Dual‐Mode L‐LECHA for EV‐miRNA Profiling

**FIGURE 4:**
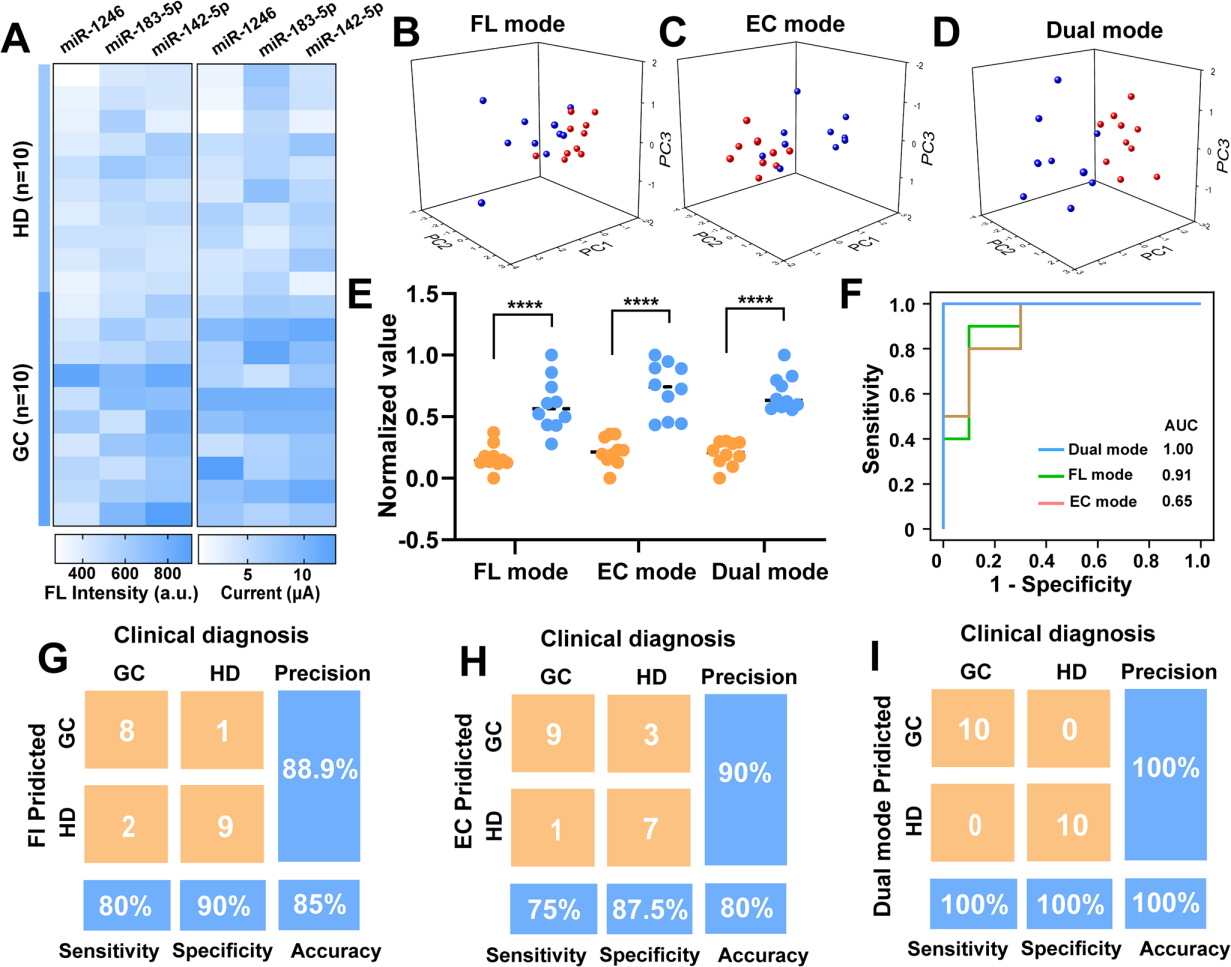
Clinical feasibility of the dual‐mode L‐LECHA for EV‐miRNA profiling. (A) The expression of the three EV‐miRNAs measured by dual‐mode L‐LECHA analysis in GC cohort (healthy donors, *n* = 10; GC patients, *n* = 10). (B‐D) PCA of GC patients and HD controls using the three EV‐miRNAs in the fluorescence platform (B), electrochemical platform (C), and dual‐mode L‐LECHA (D) as input. (E) The PCA value of fluorescence platform, electrochemical platform, and dual‐mode L‐LECHA for differentiating GC patients from HD controls. (F) The ROC analysis of fluorescence platform, electrochemical platform, and dual‐mode for discriminating GC patients and HD donors. (G‐I) The confusion matrices of GC and HD discrimination using fluorescence (G), electrochemical (H), and dual‐mode L‐LECHA (I). Statistical analysis for E was performed using unpaired t test (^****^ *p* ≦ 0.0001).

We first recruited a preliminary cohort of 10 early‐stage GC patients and 10 healthy donors to evaluate the clinical feasibility of the platform (Table S1). Figure 4A illustrated a heatmap for the relative expression of the three EV‐miRNAs in electrochemical mode and fluorescence mode. Compared with HDs, expression of the three EV‐miRNAs in GC patients was upregulated to varying degrees both in fluorescence mode and electrochemical mode. To assess the capacity for GC diagnosis, three‐dimensional principal component analysis (3D‐PCA) was employed. For the dual‐mode analysis, the three fluorescence and three electrochemical measurements were retained as six independent variables and reduced to PC1‐PC3. Thus, the 3D plot represents dimensionality reduction from six measurements to three principal components. As shown in Figure 4B–D, single‐mode L‐LECHA analysis (fluorescence mode and electrochemical mode) using the EV‐miRNA panel showed partial overlap in the 3D‐PCA plots, whereas the dual‐mode analysis showed almost complete separation, demonstrating superior discrimination capacity of the panel analysis using the proposed dual‐mode L‐LECHA platform. To further validate the diagnostic capacity of dual‐mode L‐LECHA analysis, the analysis data for the three miRNAs in fluorescence and electrochemical modes were combined using logistic regression. As shown in Figure 4E, single‐mode analyses alone were insufficient to fully distinguish HD from GC patients. In contrast, the dual‐mode analysis achieved complete separation, indicating significantly enhanced discriminatory performance of dual‐mode analysis. Subsequently, we generated receiver operating characteristic (ROC) curves for the three analytical methods (fluorescence mode, electrochemical mode, and dual‐mode combination) based on the logistic regression analysis. The dual‐mode analysis achieved a perfect AUC of 1.00, substantially outperforming individual fluorescence or electrochemical analysis (Figure 4F), as well as single‐miRNA and dual‐miRNA analyses (Figure S17). The confusion matrices also confirmed the strong diagnostic performance using the dual‐mode L‐LECHA analysis (Figure 4G–I). These results indicate the applicability of the proposed dual‐mode L‐LECHA for GC diagnosis.

### 2.5 Machine‐Learning‐Assisted Gastric Cancer Diagnosis Using Dual‐Mode L‐LECHA

**FIGURE 5:**
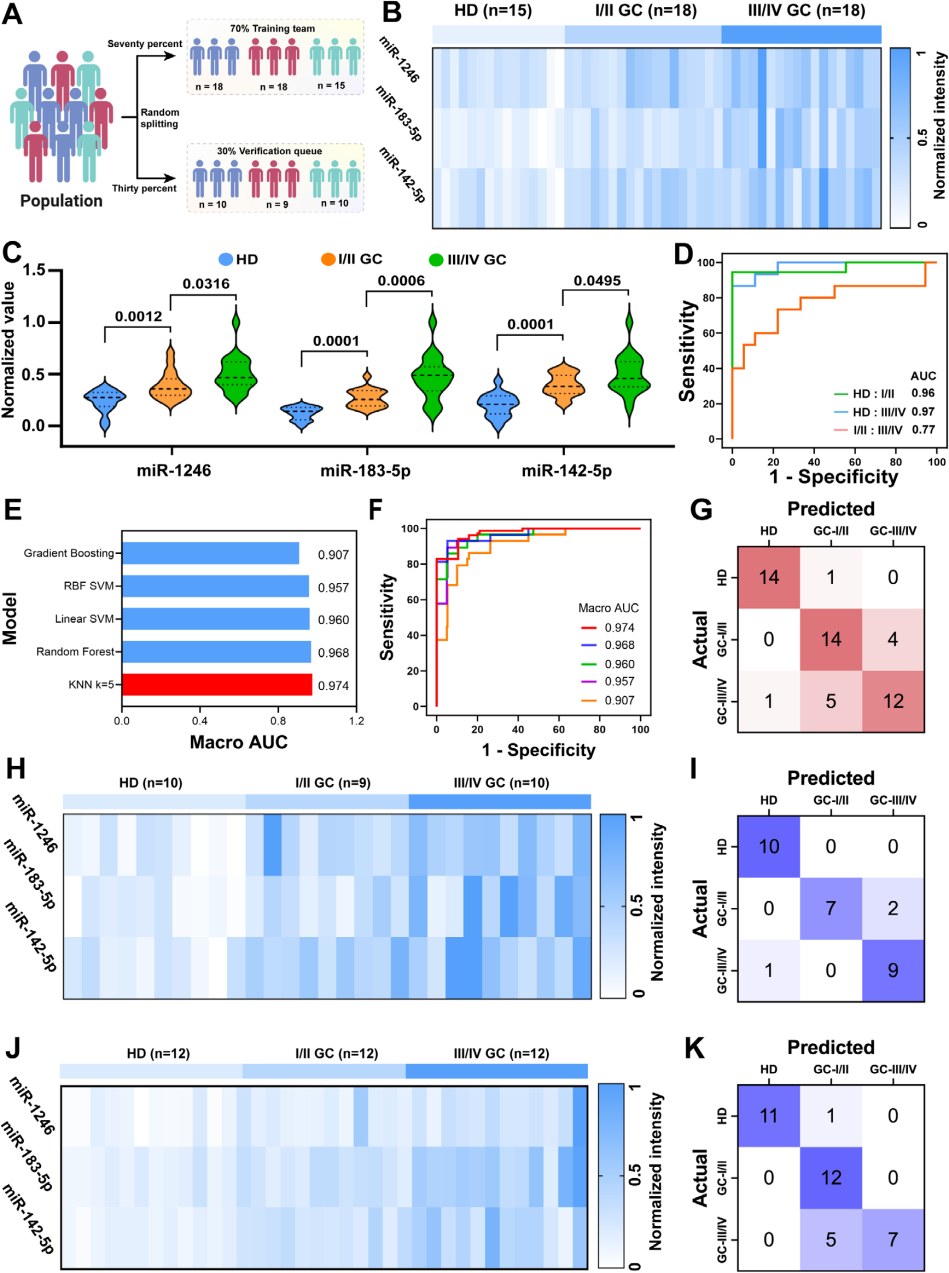
Machine‐learning‐assisted gastric cancer diagnosis using the dual‐mode L‐LECHA. (A) Schematic illustration of the cohort study design using the dual‐mode L‐LECHA platform. (B) The expression of the three EV‐miRNAs (EV‐miR‐1246, EV‐miR‐183‐5p and EV‐miR‐142‐5p) measured by dual‐mode L‐LECHA analysis in the GC training cohort (healthy donors, *n* = 15; stage I‐II, *n* = 18, and stage III‐IV, *n* = 18). (C) The dual‐mode L‐LECHA readout for EV‐miR‐1246, EV‐miR‐183‐5p, and EV‐miR‐142‐5p profiling in the GC training cohort. (D) The ROC analysis of discrimination among HD, stage I‐II and stage III‐IV by direct combination of the readout of three EV‐miRNAs using dual‐mode L‐LECHA in the training cohort. (E,F) The ROC analysis of the five ML models, including Gradient Boosting, RBF SVM, Linear SVM, Random Forest, and KNN in the training cohort. (G) The confusion matrix for discrimination among HD, stage I–II and stage III–IV using the KNN model in the training cohort. (H) The expression of the three EV‐miRNAs measured by dual‐mode L‐LECHA analysis in the GC validation cohort (healthy donors, *n* = 10; stage I‐II, *n* = 9, and stage III‐IV, *n* = 10). (I) The confusion matrix for discrimination among HD, stage I–II and stage III–IV using the KNN model in the validation cohort. (J) The expression of the three EV‐miRNAs measured by dual‐mode L‐LECHA analysis in the external validation cohort. (K) The confusion matrix for discrimination among HD, stage I–II and stage III–IV using the KNN model in the external validation cohort. Statistical analysis was performed using unpaired t‐test.

The expanded stage‐classification cohort comprised 25 healthy donors, 27 patients with stage I‐II GC, and 28 patients with stage III‐IV GC. The initial 10 healthy donors and 10 early‐stage GC patients from the preliminary feasibility analysis were included within this sequentially expanded cohort. Further, the cohort was randomly divided into training and validation cohorts at a 7:3 ratio (Figure 5A and Table S2). The training cohort (15 HD, 18 stage I–II and 18 stage III–IV) was analyzed using the dual‐mode L‐LECHA platform. The results shown in Figure 5B,C indicate varying degrees of increase in the dual‐mode‐corrected expression of the three EV‐miRNAs. The ROC analysis was then employed to validate the diagnostic capacity of the proposed platform by coupling the three miRNAs. As illustrated in Figure 5D, the direct combination of three miRNAs showed only moderate diagnostic capacity (AUC_HD: Stage I‐II_ 0.96, AUC_HD: Stage III‐IV_ 0.97 and AUC_Stage I‐II: Stage III‐IV_ 0.77). To further improve diagnostic performance, the machine learning (ML) models screened included Gradient Boosting, RBF SVM, Linear SVM, Random Forest, and KNN. As shown in Figure 5E, the KNN model showed the best accuracy (AUC = 0.974), with a stable and robust performance across the five cross‐validation folds (Figure S18). Based on the KNN model, ROC analysis showed better classification performance among HD, stage I‐II, and stage III‐IV (AUC_HD: Stage I‐II_ 0.97, AUC_HD: Stage III‐IV_ 0.99 and AUC_Stage I‐II: Stage III‐IV_ 0.84) (Figure 5F). The confusion matrix also confirmed the strong discriminative capacity of machine‐learning‐assisted dual‐mode L‐LECHA analysis with an overall accuracy of 78.43% (Figure 5G).

The locked KNN model was then evaluated without further adjustment in the 29‐sample validation subset, which comprised 10 healthy donors, 9 patients with stage I‐II GC, and 10 patients with stage III‐IV GC. The validation‐set macro‐AUC was 0.940 (95% CI, 0.886–0.983), with sensitivity of 79.26% and specificity of 88.97%. Count‐based evaluation showed that 26 of 29 samples were classified correctly, corresponding to an overall accuracy of 89.66% (Figure 5H,I and Figure S19). An additional external cohort comprising 12 healthy donors, 12 patients with stage I‐II GC, and 12 patients with stage III‐IV GC yielded an AUC of 0.9132 (95% CI, 0.832‐0.979) and an overall accuracy of 83.33% (Figure 5J,K and Figure S20 and Table S3). These results indicate that dual‐mode L‐LECHA can serve as a powerful platform for gastric cancer diagnosis. Moreover, the proposed machine‐learning‐assisted dual‐mode L‐LECHA platform could also discriminate pathological types, achieving an overall accuracy of 87% in cohort testing (Figure S21 and Table S4), indicating the versatility of the proposed platform.

## 3 Discussion

Gastric cancer remains difficult to manage clinically because reliable non‐invasive tools for early diagnosis are still limited. EV‐miRNAs are promising liquid‐biopsy biomarkers because they are protected within vesicles and may reflect tumor‐associated molecular alterations. However, their clinical translation is still restricted by several analytical bottlenecks, including target loss during EV lysis and RNA extraction, insufficient sensitivity of nonamplified in situ probes, background leakage of enzyme‐free amplification circuits, and matrix‐associated variability in plasma samples. To address these issues, we developed a liposome‐encapsulated localized exponential catalytic hairpin assembly platform, termed L‐LECHA, with the aim of integrating vesicle‐compatible probe delivery, exponential signal amplification, and dual‐mode signal readout for EV‐miRNA analysis.

L‐LECHA achieved an RNA detection limit of 52.48 aM, representing a 10.96‐fold improvement over the typical ECHA. Compared with representative EV‐miRNA detection platforms, L‐LECHA provides a favorable balance of rapid reaction time, broad analytical range, multiplex capability, in situ EV‐miRNA analysis, and complementary dual‐mode readout (Table S5). Based on this strategy, the platform can detect EV‐miRNAs in situ at an EV concentration of 954.99 particles/µL. Furthermore, when coupled with an EV‐miRNA panel and KNN machine learning model, the proposed platform could discriminate among HDs, patients with stage I–II GC, and patients with stage III–IV GC with an AUC of 0.974 and a count‐based accuracy of 78.43% (40/51) in the development cohort. The 29‐sample validation cohort yielded an overall accuracy of 89.66% (26/29), a macro‐AUC of 0.940 (95% CI, 0.886‐0.983), sensitivity of 79.26%, and specificity of 88.97%. The proposed platform could also be used to discriminate pathological types, achieving an accuracy of 87%.

Despite these promising results, the dual‐mode L‐LECHA analysis platform has several limitations. First, the workflow still requires pre‐isolation of EVs, which leads to a total processing time of more than 2 h. Future technical improvements are required to enhance isolation efficiency, such as coupling the platform with microfluidic devices to enable one‐step detection and support point‐of‐care applications. Second, the clinical performance observed in this study may not be fully representative of real‐world performance because the cohort included only 80 individuals and the validation cohort was small. We expect to conduct a large‐scale, multicenter clinical validation in the future to comprehensively evaluate the real‐world performance of the proposed platform. Moreover, combining different classes of EV biomarkers, such as miRNAs and EV‐associated proteins, may provide broader biological information, enhancing clinical application, which can be studied in the future.

In summary, we have established a dual‐mode platform for in situ detection of EV‐miRNA. Coupled with an EV‐miRNA panel and machine learning, the proposed platform could be used for GC diagnosis and pathological stratification, and could be adopted for GC liquid biopsy in the future.

## 4 Methods

### 4.1 Reagents, Materials and Instruments

All DNA and RNA oligonucleotides used in this study were synthesized and purified by BGI Genomics Co., Ltd. (Shenzhen, China). The oligonucleotides included synthetic miR‐1246, miR‐183‐5p and miR‐142‐5p targets, mismatched control sequences, random RNA controls, LECHA hairpin probes, DNA scaffold strands, fluorophore‐labeled probes, quencher‐labeled probes and thiol‐modified electrochemical capture probes. The detailed sequences and modifications are listed in Table S6.

The 500 bp DNA marker and 6× loading buffer were purchased from Accurate Biology (Hunan, China). TE buffer and 4S Green Plus nucleic acid stain were obtained from Sangon Biotech (Shanghai, China). Phosphate‐buffered saline (PBS), RPMI‐1640 medium, fetal bovine serum, EV‐depleted fetal bovine serum, and trypsin were obtained from Thermo Fisher Scientific. The TNaK hybridization buffer consisted of 125 mM NaCl, 20 mM Tris‐HCl and 20 mM KCl at pH 7.5. DOTMA, cholesterol, and DSPE‐PEG2000 were used for cationic liposome preparation. 6‐Mercapto‐1‐hexanol (MCH) and RuHex were obtained from Sigma–Aldrich. RNase A was used for nuclease‐protection experiments. Unless otherwise specified, all other reagents were of analytical grade, and all aqueous solutions were prepared using nuclease‐free water.

Fluorescence measurements were performed using a Spark microplate reader (Tecan, Switzerland). RT–qPCR analysis was performed on a LightCycler 480 system (Roche, Switzerland). Gel electrophoresis was performed using a Syngene electrophoresis system, and gel images were acquired using a Gel Doc XR imaging system (Bio‐Rad, USA). Electrochemical measurements were performed using a CHI‐660E electrochemical workstation. TEM (HITACHI H‐7650), DLS (Malvern Zetasizer Nano ZS90), and zeta‐potential analysis (Malvern Zetasizer Nano ZS90) were performed by Scientific Compass, China. Fluorescence resonance energy transfer (FRET) (Tecan, Switzerland), ultraviolet‐visible spectroscopy (Tecan, Switzerland), nanoparticle tracking analysis (NTA) (NanoSight NS300), super‐resolution fluorescence microscopy (Nikon N‐STORM) and nano‐flow cytometry (NanoFCM) (NanoAnalyzer) were used to characterize EVs, L‐LECHA probes and L‐LECHA‐treated EVs.

### 4.2 Cell Culture and EV Collection

Human gastric cancer cell lines AGS, MKN‐74 and HGC‐27 and the human gastric mucosal epithelial cell line GES‐1 were used in this study. The cell lines were purchased from Procell Life Science & Technology Co., Ltd. and cultured in RPMI‐1640 medium supplemented with 10% fetal bovine serum, 100 U/mL penicillin and 100 µg/mL streptomycin at 37°C in a humidified incubator containing 5% CO_2_. All cell lines were tested using the MycoSEQ Mycoplasma Detection Kit and confirmed to be free of mycoplasma contamination. For EV collection, cells were cultured until approximately 80% confluence, washed with PBS, and maintained in serum‐free medium for 12 h. The medium was then replaced with RPMI‐1640 medium supplemented with EV‐depleted fetal bovine serum and cultured for 36 h. The conditioned medium was collected and immediately processed for EV isolation.

### 4.3 Clinical Samples

Clinical plasma samples were obtained from Nanfang Hospital, Southern Medical University. Gastric cancer patients were included only after histopathological confirmation and before treatment (Figure S22). Healthy donors were recruited through routine physical examination and had no history of cancer or major chronic disease, including hypertension or diabetes. Participants were excluded if samples showed visible hemolysis, severe lipemia, turbidity, clot formation, insufficient volume, repeated freeze‐thaw cycles, delayed processing, prolonged improper storage, or incomplete clinical information. The first 10 early‐stage GC patients and 10 healthy donors were recruited for the preliminary proof‐of‐concept analysis and were subsequently retained in the expanded stage‐classification cohort rather than treated as an independent cohort. The expanded cohort was used for model development and validation, and an additional external cohort was recruited for independent evaluation. Written informed consent was obtained from all participants. The study was approved by the institutional ethics committee of Nanfang Hospital, Southern Medical University (NFEC‐2024‐332), and was conducted in accordance with the Declaration of Helsinki.

Peripheral blood was collected into anticoagulant tubes and processed promptly after collection. Whole blood was first centrifuged at 3000 × g for 10 min to obtain cell‐free plasma. The plasma fraction was then centrifuged at 10 000 × g for 20 min at 4°C to remove residual cells, debris, and large vesicular particles. The clarified plasma was aliquoted and stored at −80°C before EV isolation. Samples with visible hemolysis, severe lipemia, turbidity, clot formation, insufficient volume, repeated freeze–thaw cycles, delayed processing, prolonged improper storage, or incomplete clinical information were excluded from downstream analysis.

### 4.4 EV Isolation and Characterization

EVs from cell culture supernatants and clinical plasma samples were isolated using the EXODUS platform. For cell culture samples, conditioned medium was sequentially centrifuged at 3000 × g for 10 min, 8000 × g for 20 min and 10 000 × g for 30 min to remove cells, apoptotic bodies, cellular debris, and large particles. The supernatant was filtered through a 0.22 µm membrane and processed using the EXODUS system. For plasma samples, clarified plasma was processed using the same EV isolation platform. Isolated EVs were resuspended in PBS and stored at −80°C until use.

EV morphology was characterized by TEM. EV size distribution and particle concentration were measured by NTA. NanoFCM was used to analyze EV size distribution and single‐particle fluorescence signals from L‐LECHA‐treated EVs.

### 4.5 Assembly of Lecha

Before assembly, each hairpin probe was dissolved in TE buffer, heated at 95°C for 5 min, and slowly cooled to 25°C to form a stable hairpin conformation. The annealed CHA hairpins (1 µM) and DNA scaffold strands (1 µM) were then mixed in TNaK buffer and incubated at room temperature for 1 h to obtain the LECHA complex.

Agarose gel electrophoresis was used to verify LECHA assembly. Samples were mixed with 6× loading buffer, loaded onto agarose gels containing nucleic acid stain, and electrophoresed in 1× TAE buffer at 140 V for 35 min. Gel images were acquired using a gel imaging system. The formation of higher‐molecular‐weight bands was used to confirm scaffold‐mediated assembly of the LECHA complex.

### 4.6 LECHA Reaction Assay

Synthetic miR‐1246 was used as the model input strand to verify the LECHA reaction. LECHA was mixed with miR‐1246 in TNaK buffer and incubated at 25°C unless otherwise stated. Fluorescence signals were recorded at different reaction times or at the endpoint. FAM fluorescence was measured at an excitation wavelength of 488 nm and an emission wavelength of 515 nm.

For electrochemical detection, LECHA reaction products were hybridized with complementary thiolated capture probes immobilized on gold electrodes. Briefly, gold electrodes were cleaned, modified with thiol‐DNA capture probes through Au–S chemistry, and blocked with MCH to reduce nonspecific adsorption and orient the DNA layer. After hybridization with LECHA products, RuHex was added as the electroactive indicator. Differential pulse voltammetry was then performed using an electrochemical workstation.

### 4.7 Optimization of LECHA Reaction Conditions

The LECHA reaction conditions were optimized using synthetic miR‐1246 as the input. Reaction time, reaction temperature, scaffold length, and connection length were evaluated by comparing fluorescence intensity, DPV current, background signal, and signal‐to‐noise ratio. The optimized conditions were selected to balance amplification efficiency, reaction kinetics, and leakage suppression.

The trigger domains were standardized as segment 1 and segment 4 throughout the manuscript, figures, captions, and axis labels. Candidate segment 1/segment 4 combinations were evaluated using NUPACK to compare thermodynamic stability and predicted secondary structure. The complete NUPACK input strand structures, sequence‐domain definitions, and strand compositions are provided in the Supplementary Information (Table S7). The final design was selected by jointly considering target‐triggered signal output, background leakage, and signal‐to‐noise ratio.

### 4.8 Analytical Performance of Lecha

The sensitivity of LECHA was evaluated using synthetic miR‐1246 standards ranging from 10 aM to 10 nM. Fluorescence and DPV signals were recorded after the LECHA reaction. Calibration curves were generated by plotting fluorescence intensity or DPV current against target concentration. The limit of detection was calculated according to the background plus three standard deviations criterion, and the corresponding target concentration was obtained from the calibration curve.

The specificity of LECHA was evaluated using perfectly matched miR‐1246, single‐base mismatched targets, random RNA controls, and noncorresponding miRNA targets. All targets were tested under the same reaction conditions. Specificity was determined by comparing the signal generated by the perfectly matched target with the signals generated by mismatched and nontarget controls.

### 4.9 Comparison of LECHA With Other CHA Systems

To evaluate the contribution of localized exponential amplification, LECHA was compared with localized cascade CHA, exponential CHA without scaffold localization, and scaffold‐localized all‐exponential CHA. Each system was triggered by miR‐1246 under identical reaction conditions. Reaction kinetics, fluorescence intensity, DPV current, background leakage, and signal‐to‐noise ratio were compared. These experiments were used to determine whether the LECHA architecture provided a better balance between amplification efficiency and background suppression than the alternative CHA designs.

### 4.10 Preparation of L‐LECHA

L‐LECHA was prepared using the rotary evaporation method. DOTMA, cholesterol, and DSPE‐PEG2000 were dissolved in chloroform and mixed at a molar ratio of 2:1:1. The organic solvent was removed by rotary evaporation at 35°C to form a thin lipid film. Preassembled LECHA solution was added to hydrate the lipid film in TNaK buffer. The mixture was subjected to ultrasonic hydration at 37°C for 1 h with intermittent cooling to generate liposome‐encapsulated LECHA probes. Free LECHA probes were removed by ultrafiltration. The resulting L‐LECHA probes were stored at 4°C before use. NTA measured EV and L‐LECHA concentrations of 1.01 × 10^9^ and 2.47 × 10^9^ particles/mL, respectively, corresponding to an EV:L‐LECHA particle ratio of approximately 1:2.46 under the reported assay conditions. The total and unencapsulated LECHA concentrations were 28.5 and 9.3667 ng/µL, respectively, yielding an encapsulation efficiency of 67.13%. Based on the total number of L‐LECHA particles, the average loading was estimated to be approximately 55 LECHA molecules per particle. Storage stability was evaluated during refrigerated storage by analytical response at the specified time points.

The morphology of L‐LECHA was characterized by TEM. The hydrodynamic diameter was measured by DLS, and the surface charge was measured by zeta‐potential analysis. These characterizations were used to confirm the successful preparation of cationic liposome‐encapsulated LECHA probes.

### 4.11 Fusion of L‐LECHA With EVs

AGS‐derived EVs were used to evaluate interactions between L‐LECHA and EVs. Under the reported assay conditions, EVs and L‐LECHA were used at particle concentrations of 1.01 × 10^9^ and 2.47 × 10^9^ particles/mL, respectively, corresponding to an EV:L‐LECHA ratio of approximately 1:2.46. Changes in hydrodynamic diameter and surface charge after incubation were measured by DLS and zeta‐potential analysis, and FRET and ultraviolet–visible spectroscopy provided complementary membrane‐interaction evidence. Because size and charge shifts can also reflect adsorption or aggregation, these measurements were not interpreted alone as definitive evidence of complete fusion. Parallel control experiments compared cationic DOTMA, neutral DOPC, and anionic DPPG liposomes under identical conditions.

For orthogonal fluorescence characterization, EVs were labeled with a Cy5‐conjugated anti‐CD63 antibody, and liposomes carried FAM‐labeled DNA probes. Super‐resolution confocal microscopy was used to assess spatial colocalization of the EV‐associated CD63 signal with the liposome‐derived cargo signal. NanoFCM independently identified CD63‐positive EV events and probe‐positive cargo events; double‐positive particles were defined using the corresponding single‐color and unlabeled controls.

### 4.12 In Situ EV‐miRNA Detection Using L‐LECHA

Purified EVs were incubated with L‐LECHA under the optimized reaction conditions. Target recognition and LECHA amplification occurred while EVs remained intact. For fluorescence‐mode detection, the signal was measured directly after the intravesicular reaction without EV disruption. For electrochemical‐mode detection, a parallel reacted aliquot was lysed using 0.1% triton‐100 for 30 min only after the *intravesicu*lar reaction had been completed; the released amplification products were then collected for DPV measurement. Accordingly, the workflow is described as an in situ intravesicular reaction followed by post‐reaction electrochemical detection, rather than direct in situ electrochemical measurement.

AGS EVs and GES‐1 EVs were used to validate L‐LECHA‐based in situ detection of EV‐miR‐1246. Treated EVs were analyzed by super‐resolution fluorescence microscopy, NanoFCM, bulk fluorescence measurement, and DPV measurement. Signals from AGS EVs and GES‐1 EVs were compared to evaluate whether L‐LECHA could distinguish EV‐miRNA expression differences between gastric cancer cells and non‐malignant gastric epithelial cells.

### 4.13 RNase Protection Assay

To compare the intravesicular and pre‐lysis workflows, EVs were analyzed with and without FBS treatment. For the intravesicular workflow, intact EVs were incubated with L‐LECHA before signal collection. For the pre‐lysis workflow, EVs were disrupted before the LECHA reaction.

### 4.14 Cell‐Line EV‐miRNA Profiling

EVs isolated from AGS, MKN‐74, HGC‐27 and GES‐1 cells were adjusted to the same particle concentration before analysis. L‐LECHA probes targeting miR‐1246, miR‐183‐5p and miR‐142‐5p were used for EV‐miRNA profiling. Fluorescence signals were recorded after L‐LECHA treatment, and electrochemical signals were recorded by DPV after collection of the reaction products.

Super‐resolution fluorescence microscopy was used to visualize miR‐1246 signals in EVs from the four cell lines. Serial dilutions of EVs from each cell line were analyzed to evaluate quantitative reliability. Linear correlations between EV concentration and fluorescence or electrochemical signal were calculated.

### 4.15 Orthogonal Detection of Three EV‐miRNAs

L‐LECHA probes targeting miR‐1246, miR‐183‐5p, and miR‐142‐5p were used to evaluate orthogonal miRNA recognition. Each L‐LECHA probe was incubated with its corresponding target and non‐corresponding targets under identical conditions. Fluorescence and electrochemical signals were collected, and the resulting signal matrix was used to assess cross‐reactivity among the three miRNA assays.

### 4.16 RT–qPCR Validation

Total RNA was extracted from purified EVs using a miRNA extraction kit. Reverse transcription was performed using a miRNA reverse transcription kit, and quantitative PCR was performed using a SYBR Green‐based qPCR system. EV‐miR‐1246, EV‐miR‐183‐5p, and EV‐miR‐142‐5p were quantified. RT–qPCR results were normalized and compared with fluorescence‐mode and electrochemical‐mode signals obtained from dual‐mode L‐LECHA analysis.

### 4.17 Clinical Sample Detection Protocol

For clinical EV‐miRNA detection, clarified plasma samples were first subjected to EV isolation using the EXODUS platform as described above. The isolated plasma EVs were resuspended in PBS and then transferred into TNaK buffer for L‐LECHA analysis. For each clinical sample, EV‐miR‐1246, EV‐miR‐183‐5p and EV‐miR‐142‐5p were measured using the corresponding L‐LECHA probes under identical reaction conditions. For each target, fluorescence and electrochemical measurements were performed on parallel aliquots from the same EV preparation at an identical particle concentration. A 20 µL aliquot was used for fluorescence measurement, and a 5 µL aliquot was used for electrochemical measurement.

Plasma EVs were incubated with L‐LECHA to permit membrane interaction and intravesicular delivery of the LECHA circuit. Target EV‐miRNAs then triggered localized exponential‐like catalytic hairpin assembly within intact EVs. Fluorescence‐mode detection was performed directly after th*is* reaction using a 20 µL aliquot, and fluorescence intensity was recorded for each EV‐miRNA target without post‐reaction lysis.

For electrochemical‐mode detection, a parallel 5 µL aliquot from the fluorescence measurement was lysed after completion of the intravesicular reaction to release the amplification products. The products were hybridized with thiol‐DNA‐modified gold electrodes, followed by RuHex incubation and DPV measurement. For each EV‐miRNA, the fluorescence intensity and DPV current were first background‐corrected by subtracting their corresponding blank signals. The modality‐specific calibration relationships shown in Figure 1O were then used to convert the corrected readings to a common dimensionless scale. The concentration range from 100 aM to 10 nM was normalized on a log10 scale. Binary logistic regression was then used to integrate the two normalized readings for each miRNA, converting six mode‐specific measurements into three dual‐mode corrected variables corresponding to EV‐miR‐1246, EV‐miR‐183‐5p, and EV‐miR‐142‐5p.

### 4.18 Clinical EV‐miRNA Profiling

Plasma EVs from gastric cancer patients and healthy donors were analyzed using L‐LECHA probes targeting EV‐miR‐1246, EV‐miR‐183‐5p, and EV‐miR‐142‐5p. Each target was measured in fluorescence and electrochemical modes using parallel aliquots at the same EV particle concentration. Raw fluorescence intensities and DPV currents were background‐corrected and normalized as described above. For integrated analyses, the two normalized values for each miRNA were combined by binary logistic regression to generate three dual‐mode corrected variables. The six original measurements were retained separately for analyses that explicitly compared the two modes.

For the preliminary feasibility cohort, heatmaps were generated from the mode‐specific and integrated EV‐miRNA values. In the dual‐mode 3D PCA, the three fluorescence and three electrochemical measurements were used as six independent inputs, and PC1‐PC3 were displayed; therefore, the plot reduced six original variables to three principal components. Logistic regression combined the three miRNA markers within fluorescence‐only, electrochemical‐only, and integrated dual‐mode analyses. ROC curves and count‐based confusion matrices were used to evaluate diagnostic performance.

### 4.19 Machine Learning‐Assisted Gastric Cancer Diagnosis

For stage‐stratified gastric cancer diagnosis, dual‐mode corrected values of EV‐miR‐1246, EV‐miR‐183‐5p, and EV‐miR‐142‐5p were used as input features. The development subset comprised 15 healthy donors, 18 patients with stage I‐II GC, and 18 patients with stage III‐IV GC. A separate 29‐sample validation subset comprised 10 healthy donors, 9 patients with stage I‐II GC, and 10 patients with stage III‐IV GC. All preprocessing and normalization parameters were estimated within the development data and applied unchanged to held‐out samples.

Gradient Boosting, radial‐basis‐function SVM, linear SVM, Random Forest, and KNN were evaluated within the development subset. Hyperparameters were optimized by five‐fold cross‐validation. In each iteration, four folds were used for model fitting and one fold served as the held‐out test fold, so every development sample received one out‐of‐fold prediction. For KNN, candidate k values were compared and k = 5 was selected according to mean cross‐validation performance. Model performance was assessed using macro‐AUC, sensitivity, specificity, and count‐based confusion matrices.

After model selection, the preprocessing pipeline, model architecture, and KNN hyperparameters were locked. The finalized model was then applied to the validation subset without further adjustment. The validation‐set macro‐AUC was 0.940 (95% CI, 0.886‐0.983), with sensitivity of 79.26% and specificity of 88.97%. Count‐based confusion matrices and sample‐level predictions were used to calculate accuracy. An additional external cohort was evaluated using the same locked analysis workflow.

### 4.20 Machine Learning‐Assisted Pathological Classification

For pathological subtype prediction, plasma EVs from gastric cancer patients with different pathological types were analyzed using the same dual‐mode L‐LECHA workflow. EV‐miR‐1246, EV‐miR‐183‐5p, and EV‐miR‐142‐5p were measured in fluorescence mode and electrochemical mode, and the dual‐mode corrected values were used as input features.

Samples were divided into training and validation cohorts. A machine learning classifier was trained using the training cohort and evaluated using the validation cohort. ROC curves and confusion matrices were generated to evaluate pathological classification performance. Overall accuracy was calculated from the confusion matrix.

### 4.21 Data Processing and Statistical Analysis

Statistical analyses were performed using GraphPad Prism and Python‐based machine learning workflows. Data are presented as the mean ± standard deviation unless otherwise indicated. Fluorescence intensities and DPV currents were background‐corrected before normalization. Fluorescence‐mode values, electrochemical‐mode values, and integrated dual‐mode values were analyzed separately when indicated. All statistical tests were two‐sided unless otherwise specified. The number and type of replicates, definition of error bars, and statistical test are reported in the corresponding figure legends.

## Author Contributions

**Wenbin Li**: conceptualization, methodology, validation, formal analysis, investigation, data curation, visualization, writing the original draft. **Rui Fan**: methodology, validation, formal analysis, investigation, data curation, visualization. **Kun Xie**: validation, formal analysis, investigation, data curation, visualization. **Jiehua Zhong**: validation, formal analysis, investigation, data curation, visualization. **Yitong Zhu**: investigation. **Tingting Ji**: investigation, resources. **Yuanyuan Qin**: investigation. **Shijin Peng**: investigation. **Yu Zhang**: investigation. **Yuhang Guo**: investigation. **Tiange Zhang**: investigation. **Chunchen Liu**: investigation. **Bo Li**: investigation. **Lei Zheng**: conceptualization, supervision, writing, review & editing. **Ye Zhang**: conceptualization, writing the original draft, writing, review & editing, supervision, funding acquisition. X.Y: conceptualization, supervision, writing, review & editing, funding acquisition.

## Funding

This study was supported by the National Natural Science Foundation of China (U25C2045, 82230080, 82272424, 82372343, 82572682), the Guang Dong Basic and Applied Basic Research Foundation of China (2023B1515230007, 2023A1515010909), National Science Fund for Distinguished Young Scholars Cultivation Program of Nanfang Hospital, Southern Medical University (2024J004), Guangdong Provincial Clinical Research Center for Laboratory Medicine (2023B110008).

## Conflicts of Interest

The authors declare no conflicts of interest.

## Supporting information




**Supporting File**: advs77483‐sup‐0001‐SuppMat.docx.

## Data Availability

The data that support the findings of this study are available from the corresponding author upon reasonable request.
