# Supplementary material for: A Dual‐Mode In Situ EV‐miRNA Profiling Platform for Machine‐Learning‐Assisted Gastric Cancer Liquid Biopsy
Source: Adv Sci (Weinh). 2026 Sep 6:e77483. Online ahead of print. doi: 10.1002/advs.77483 (PMC13547540; doi:10.1002/advs.77483)
Supplement: Supplementary file 1 [file ADVS-9999-e77483-s001.docx]

**Supplementary information for**

**A Dual-Mode *In Situ* EV-miRNA Profiling Platform for Machine-Learning-Assisted Gastric Cancer Liquid Biopsy**

Wenbin Li^1,2&^, Rui Fan^1&^, Kun Xie^2&^, Jiehua Zhong^1&^, Yitong Zhu^1,2^, Tingting Ji^1^, Yuanyuan Qin^1,2^, Shijin Peng^1,2^, Yu Zhang^1,2^, Yuhang Guo^1^, Tiange Zhang^1^, Chunchen Liu^1,3,4^, Bo Li^1,3,4^, Lei Zheng^1,3,4,5^*, Ye Zhang^1,3,4^*, and Xiaohui Yan^1,2^*.

^1^Department of Laboratory Medicine, Nanfang Hospital, Southern Medical University, Guangzhou, 510515, P.R. China.

^2^Medical Research Center of Nanfang Hospital, Nanfang Hospital, Guangdong Provincial Key Laboratory of Proteomics, School of Basic Medical Sciences, Southern Medical University, Guangzhou, 510515, P.R. China.

^3^Guangdong Provincial Key Laboratory of Precision Medical Diagnostics, Guangdong Engineering and Technology Research Center for Rapid Diagnostic Biosensors, Guangdong Provincial Key Laboratory of Single-cell and Extracellular Vesicles, Guangdong Engineering Research Center of Advanced In Vitro Diagnostic Technology, Nanfang Hospital, Southern Medical University, Guangzhou, 510515, P. R. China.

^4^Guangdong Provincial Clinical Research Center for Laboratory Medicine.

^5^State Key Laboratory of Multi-organ Injury Prevention and Treatment, Southern Medical University; National Clinical Research Center for Kidney and Urological Diseases, Nanfang Hospital, Southern Medical University, Guangzhou, 510515, P.R. China.

^&^These authors contributed equally.

*Corresponding authors, gzyanxh@smu.edu.cn (Xiaohui Yan); zhangye232@i.smu.edu.cn (Ye Zhang); nfyyzhenglei@smu.edu.cn (Lei Zheng).

**1. Supplementary figures**

**Supplementary Fig. 1.** Calculation of the effective reaction volume and theoretical localized concentration of LECHA. The non-localized substrate concentration was C = 100 nM = 1.00*10^-7 mol/L. According to V = 1/(N*C), where N = 6.022*10^23 mol^-1, the average volume available per substrate molecule in the non-localized system was V = 1/[(6.022*10^23)*(1.00*10^-7)] = 1.66*10^-17 L. For LECHA, the scaffold-defined distance between the reacting substrates was estimated to be 28 bp. Using an axial rise of 0.34 nm per base pair, the effective reaction radius was r = 28*0.34 = 9.52 nm = 9.52*10^-9 m. Assuming a spherical reaction space, the effective reaction volume was V_LECHA_ = 4*π*r^3/3 = 4*π*(9.52*10^-9)^3/3 = 3.61*10^-24 m^3 = 3.61*10^-21 L. The corresponding theoretical localized concentration was C_LECHA_ = 1/(NA*V_LECHA_) = 1/[(6.022*10^23)*(3.61*10^-21)] = 4.60*10^-4 mol/L = 4.60*10^5 nM. Therefore, LECHA produced a theoretical localization enhancement of C_LECHA_/C = (4.60*10^5)/100 = 4.60*10^3-fold relative to the non-localized system. This value represents a theoretical effective concentration based on a simplified spherical-confinement model rather than a directly measured solution concentration.

**Supplementary Fig. 2**. The scheme of the LCHA reaction.

**Supplementary Fig. 3.** Electrochemical characterization of the LECHA sensing interface. (A) Electrochemical impedance spectra, (B) differential pulse voltammetry (DPV) curves and (C) cyclic voltammetry (CV) curves recorded during stepwise electrode construction. In all panels, curve a represents the bare gold electrode, curve b represents the thiolated capture-probe-modified gold electrode, curve c represents the MCH-blocked electrode, and curve d represents the electrode after hybridization with the LECHA reaction product.

**Supplementary Fig. 4**. 2% agarose gel electrophoresis analysis of LECHA reaction.

**Supplementary Fig. 5.** Comparison of the theoretical localization effects of ECHA, LECHA, and LAECHA. For non-localized ECHA at C_0_ = 100 nM = 1.00*10^-7 mol/L, the average volume available per substrate molecule was V_ECHA_ = 1/(N*C_0_) = 1/[(6.022*10^23)*(1.00*10^-7)] = 1.66*10^-17 L, and the concentration enhancement was defined as 1. In LECHA, each CHA module was locally organized by a DNA scaffold. A substrate separation of 28 bp corresponded to an effective radius of r = 28*0.34 = 9.52 nm. Assuming a spherical reaction space, the first-level effective reaction volume was V_LECHA_ = 4*π*r^3/3 = 4*π*(9.52*10^-9)^3/3 = 3.61*10^-24 m^3 = 3.61*10^-21 L. The corresponding theoretical localized concentration was C_LECHA_ = 1/(N*V_LECHA_) = 1/[(6.022*10^23)*(3.61*10^-21)] = 4.60*10^-4 mol/L = 4.60*10^5 nM. Thus, the first-level localization factor relative to 100 nM ECHA was alpha = C_LECHA_/C0 = (4.60*10^5)/100 = 4.60*10^3. In LAECHA, all CHA hairpins from the two already localized amplification modules were further confined on the same DNA nanoscaffold. This secondary confinement was modeled as a second equivalent localization step with the same dimensionless localization factor alpha. The compounded equivalent reaction volume was therefore V_LAECHA_ = V_LECHA_/alpha = (3.61*10^-21)/(4.60*10^3) = 7.87*10^-25 L. The corresponding apparent compounded effective concentration was C_LAECHA_ = 1/(N*V_LAECHA_) = 1/[(6.022*10^23)*(7.87*10^-25)] = 2.11 mol/L = 2.11*10^9 nM. The overall theoretical enhancement relative to 100 nM ECHA was C_LAECHA_/C0 = (2.11*10^9)/100 = 2.11*10^7-fold. The LAECHA value is an equivalent compounded effective concentration describing two successive confinement steps and should not be interpreted as a directly measured solution concentration or as the volume of a literal molecular sphere.

**Supplementary Fig. 6.** Dual-mode calibration analysis of the local ECHA structure with different matrix positioning designs.(A) Fluorescence spectra of ECHA's response to different concentrations of miR-1246 and corresponding calibration curves. Curves a–f correspond to miR-1246 concentrations of 10 nM, 1 nM, 100 pM, 10 pM, 1 pM, and 100 fM, respectively.(B) DPV response of ECHA to different concentrations of miR-1246 and corresponding calibration curves. Curves a–f correspond to miR-1246 concentrations of 100 pM, 10 pM, 1 pM, 100 fM, 10 fM, and 1 fM, respectively.(C) Fluorescence spectra of CHA's response to different concentrations of miR-1246 and corresponding calibration curves. Curves a–e correspond to miR-1246 concentrations of 10 nM, 1 nM, 100 pM, 10 pM, and 1 pM, respectively.(D) DPV response of CHA to different concentrations of miR-1246 and corresponding calibration curves. Curves a–e correspond to miR-1246 concentrations of 100 pM, 10 pM, 1 pM, 100 fM, and 10 fM, respectively. The inset in the figure shows the corresponding logarithmic linear calibration curves, and the dotted line represents the blank value + 3SD threshold used for sensitivity assessment.

**Supplementary Fig. 7** Structural optimization of the scaffold and connection lengths in the LECHA system. (A,B) Fluorescence intensity and DPV current obtained using different scaffold lengths of 5, 10, 15 and 20 bp.

**Supplementary Fig. 8** (A) Time-dependent dual-mode response of LECHA at 15, 25, 35, 45 and 55 min, with DPV current shown as bars and fluorescence intensity shown as the line plot. (B) Temperature-dependent dual-mode response of LECHA at 4, 25, 37, 45 and 60 degrees C, with DPV current shown as bars and fluorescence intensity shown as the line plot.

**Supplementary Fig. 9**. (a) NTA particle counts for EV and L-LECHA preparations. (b) EV:L-LECHA particle ratio under the assay conditions. (c) Total input DNA and DNA recovered in the ultrafiltration filtrate, used to estimate encapsulated DNA. Encapsulation efficiency and mean molecules per particle are reported as mean ± SD from [n] independent preparations and should be interpreted as recovery-based estimates.

**Supplementary Fig. 10** Characterization of EVs isolated from AGS cells. (A) TEM images showing vesicular morphology of isolated EVs. (B) Size distribution and particle concentration measured by nanoparticle tracking analysis. (C) Representative EV marker analysis of TSG101, CD9 and CD63.

**Supplementary Fig. 11** UV-vis absorbance spectra of EVs, liposomes and liposome-EV complexes used to verify membrane fusion between L-LECHA and EVs.

**Supplementary Fig. 12**. Super-resolution confocal microscopy images and representative NanoFCM plots characterizing CD63-positive EVs and liposome-derived fluorescent cargo after incubation.

**Supplementary Fig. 13**. Super-resolution confocal microscopy images and corresponding NanoFCM results characterizing the interaction between EVs and L-LECHA prepared with lipids of different surface charges.

**Supplementary Fig. 14** Comparison of the intravesicular L-LECHA workflow and the pre-lysis LECHA workflow for EV-miR-1246 analysis with or without FBS.

**Supplementary Fig. 15**. Volcano plot showing 49 differentially expressed miRNAs between the GC and HD groups in GEO dataset GSE332730, and a heatmap showing the expression patterns of the dysregulated miRNAs.

**Supplementary Fig. 16** Orthogonal recognition of three EV-miRNA targets using L-LECHA in electrochemical mode. Heatmap of DPV currents generated by L-LECHA probes targeting miR-1246, miR-183-5p and miR-142-5p in response to matched and non-matched miRNA targets.

**Supplementary Fig. 17** Diagnostic evaluation of individual and combined EV-miRNA markers in the preliminary clinical cohort. (A) Heatmap of normalized dual-mode L-LECHA signals for EV-miR-1246, EV-miR-183-5p and EV-miR-142-5p in GC patients (n = 10) and healthy donors (n = 10). (B,C) ROC curves for individual miRNAs and multi-miRNA combinations in single-mode analysis. (D) ROC curves comparing the dual-mode three-miRNA panel with CEA and CA199 for GC discrimination.

**Supplementary Fig. 18**. Five-fold cross-validation performance in the development cohort, including ROC curves, sensitivity, and specificity.

**Supplementary Fig. 19**. ROC analysis of the KNN model in the validation set.

**Supplementary Fig. 20**. ROC analysis of the KNN model in the external validation set.

**Supplementary Fig. 21** Machine learning-assisted pathological subtype classification of GC using dual-mode L-LECHA. (A) Heatmap of normalized EV-miRNA signals across intestinal-type, diffuse-type and mixed-type GC samples. (B) Linear discriminant analysis score plot for pathological subtype separation with representative histological images. (C) ROC analysis for pathological subtype classification. (D) Confusion matrix showing pathological classification performance, with an overall accuracy of 87%.

**Supplementary Fig. 22** The overview of the clinical cohort and the processing in this study.

**2. Supplementary tables**

Table S1: Clinical information for healthy donors (HD) and gastric cancer patients (GC).

| Sample ID | Age | Stage | Sex | Diagnosis |
| --- | --- | --- | --- | --- |
| HD 1 | 57 | - | Male | - |
| HD 2 | 25 | - | Female | - |
| HD 3 | 17 | - | Male | - |
| HD 4 | 36 | - | Female | - |
| HD 5 | 57 | - | Male | - |
| HD 6 | 28 | - | Male | - |
| HD 7 | 38 | - | Female | - |
| HD 8 | 42 | - | Male | - |
| HD 9 | 32 | - | Male | - |
| HD 10 | 54 | - | Female | - |
| GC 1 | 45 | I | Female | Antral mid-low differentiated adenocarcinoma |
| GC 2 | 56 | II | Male | Fundus malignancy |
| GC 3 | 41 | I | Male | Body carcinoma |
| GC 4 | 47 | II | Female | Antral carcinoma |
| GC 5 | 55 | II | Female | Body carcinoma |
| GC 6 | 45 | I | Male | Antral low differentiated adenocarcinoma |
| GC 7 | 49 | I | Male | Body mid-low differentiated adenocarcinoma |
| GC 8 | 59 | I | Female | Body low differentiated adenocarcinoma |
| GC 9 | 74 | II | Male | Gastric malignancy |
| GC 10 | 57 | II | Male | Antral carcinoma |

Table S2: Clinical information for healthy donors (HD), stage I/II gastric cancer patients (GC I/II) and stage III/IV gastric cancer patients (GC III/IV)

| Patient ID | Age | Stage | Sex | Diagnosis |
| --- | --- | --- | --- | --- |
| HD 1 | 57 | - | Male | - |
| HD 2 | 25 | - | Female | - |
| HD 3 | 17 | - | Male | - |
| HD 4 | 36 | - | Female | - |
| HD 5 | 57 | - | Male | - |
| HD 6 | 28 | - | Male | - |
| HD 7 | 38 | - | Female | - |
| HD 8 | 42 | - | Male | - |
| HD 9 | 32 | - | Male | - |
| HD 10 | 54 | - | Female | - |
| HD 11 | 32 | - | Male | - |
| HD 12 | 44 | - | Female | - |
| HD 13 | 52 | - | Male | - |
| HD 14 | 44 | - | Male | - |
| HD 15 | 46 | - | Female | - |
| GC 1 | 45 | I | Female | Antral mid-low differentiated adenocarcinoma |
| GC 2 | 56 | II | Male | Fundus malignancy |
| GC 3 | 41 | I | Male | Body carcinoma |
| GC 4 | 47 | II | Female | Antral carcinoma |
| GC 5 | 55 | II | Female | Body carcinoma |
| GC 6 | 45 | I | Male | Antral low differentiated adenocarcinoma |
| GC 7 | 49 | I | Male | Body mid-low differentiated adenocarcinoma |
| GC 8 | 59 | I | Female | Body low differentiated adenocarcinoma |
| GC 9 | 74 | II | Male | Gastric malignancy |
| GC 10 | 57 | II | Male | Antral carcinoma |
| GC 11 | 71 | I | Male | Body mid-low differentiated adenocarcinoma |
| GC 12 | 64 | II | Male | Esophagostomach junction malignancy |
| GC 13 | 71 | I | Female | Body ring carcinoma |
| GC 14 | 61 | II | Female | Esophagopylorus fundus carcinoma |
| GC 15 | 52 | I | Male | Angular low differentiated adenocarcinoma |
| GC 16 | 50 | I | Female | Antral high differentiated adenocarcinoma |
| GC 17 | 64 | I | Female | Antral carcinoma |
| GC 18 | 66 | II | Female | Gastric malignancy |
| GC 19 | 23 | IV | Male | Pylorus fundus carcinoma |
| GC 20 | 58 | III | Male | Esophagostomach junction low differentiated adenocarcinoma |
| GC 21 | 69 | IV | Male | Antral malignancy |
| GC 22 | 45 | III | Male | Gastric malignancy |
| GC 23 | 67 | III | Male | Fundus pylorus mid differentiated adenocarcinoma |
| GC 24 | 55 | IV | Male | Fundus pylorus mid differentiated adenocarcinoma |
| GC 25 | 44 | IV | Female | Body malignancy (gastric ring carcinoma |
| GC 26 | 51 | III | Male | Fundus body low differentiated adenocarcinoma with bleeding |
| GC 27 | 39 | IV | Female | Body ring carcinoma |
| GC 28 | 55 | III | Female | Body low differentiated adenocarcinoma |
| GC 29 | 61 | IV | Male | Gastric body carcinoma |
| GC 30 | 67 | IV | Male | Antral low differentiated adenocarcinoma |
| GC 31 | 66 | III | Female | Pylorus fundus carcinoma |
| GC 32 | 37 | IV | Female | Antral adenocarcinoma |
| GC 33 | 54 | III | Male | Gastric malignant adenocarcinoma |
| GC 34 | 78 | IV | Male | Lower body greater curvature ring carcinoma |
| GC 35 | 64 | III | Male | Esophagostomach junction neuroendocrine carcinoma |
| GC 36 | 62 | III | Male | Antral carcinoma |

Table S3: Clinical information for healthy donors (HD), stage I/II gastric cancer patients (GC I/II) and stage III/IV gastric cancer patients (GC III/IV) in the external validation cohort.

| Patient ID | Age | Stage | Sex | Diagnosis |
| --- | --- | --- | --- | --- |
| HD 1 | 47 | - | Male | - |
| HD 2 | 88 | - | Male | - |
| HD 3 | 82 | - | Male | - |
| HD 4 | 71 | - | Female | - |
| HD 5 | 52 | - | Male | - |
| HD 6 | 72 | - | Male | - |
| HD 7 | 57 | - | Male | - |
| HD 8 | 75 | - | Female | - |
| HD 9 | 58 | - | Female | - |
| HD 10 | 78 | - | Male | - |
| HD 11 | 63 | - | Female | - |
| HD 12 | 47 | - | Male | - |
| GC 1 | 73 | II | Female | Well-to-moderately differentiated adenocarcinoma |
| GC 2 | 60 | II | Male | Gastric antral cancer |
| GC 3 | 63 | II | Male | Gastric antral cancer |
| GC 4 | 66 | I | Female | Cancer of the gastric cardia and fundus |
| GC 5 | 64 | II | Male | Poorly differentiated gastric adenocarcinoma |
| GC 6 | 76 | II | Female | Gastric malignant tumor |
| GC 7 | 62 | II | Male | Poorly differentiated adenocarcinoma of the gastric angle |
| GC 8 | 57 | I | Female | Poorly differentiated adenocarcinoma of the gastric body |
| GC 9 | 81 | I | Male | Gastric stump cancer |
| GC 10 | 64 | II | Male | Cancer of the gastric antrum and body |
| GC 11 | 39 | I | Male | Moderately-to-poorly differentiated adenocarcinoma |
| GC 12 | 46 | I | Male | Malignant tumor of the gastric antrum and body |
| GC 13 | 44 | IV | Female | Adenocarcinoma of the gastric fundus and body |
| GC 14 | 61 | III | Male | Moderately-to-poorly differentiated adenocarcinoma of the gastric angle |
| GC 15 | 62 | III | Female | Gastric antral cancer |
| GC 16 | 43 | IV | Male | Gastric antral cancer |
| GC 17 | 50 | III | Female | Poorly differentiated adenosquamous carcinoma |
| GC 18 | 58 | IV | Female | Signet-ring cell carcinoma |
| GC 19 | 88 | IV | Male | Moderately differentiated adenocarcinoma of the gastric body |
| GC 20 | 56 | III | Male | Gastric antral cancer |
| GC 21 | 77 | IV | Female | Cancer of the gastric fundus and body |
| GC 22 | 73 | IV | Male | Cancer of the gastric cardia and fundus |
| GC 23 | 81 | IV | Female | Moderately differentiated adenocarcinoma of the gastric fundus |
| GC 24 | 71 | III | Female | Poorly differentiated adenocarcinoma of the gastric angle with bleeding |

Table S4: Clinical information for Intestinal-type gastric cancer, diffuse-type gastric cancer and mixed-type gastric cancer.

| Sample ID | Age | Sex | Typle |
| --- | --- | --- | --- |
| 1 | 69 | Male | Intestinal-type |
| 2 | 44 | Male | Intestinal-type |
| 3 | 44 | Male | Intestinal-type |
| 4 | 49 | Female | Intestinal-type |
| 5 | 68 | Male | Intestinal-type |
| 6 | 61 | Female | Intestinal-type |
| 7 | 72 | Male | Intestinal-type |
| 8 | 58 | Male | Intestinal-type |
| 9 | 78 | Female | Intestinal-type |
| 10 | 69 | Female | Intestinal-type |
| 11 | 52 | Male | Intestinal-type |
| 12 | 55 | Male | Intestinal-type |
| 13 | 54 | Male | Intestinal-type |
| 14 | 73 | Female | Intestinal-type |
| 15 | 54 | Male | Intestinal-type |
| 16 | 54 | Male | Intestinal-type |
| 17 | 38 | Male | Intestinal-type |
| 18 | 77 | Male | Intestinal-type |
| 19 | 60 | Male | Intestinal-type |
| 20 | 47 | Female | Intestinal-type |
| 21 | 64 | Female | Intestinal-type |
| 22 | 56 | Female | Intestinal-type |
| 23 | 52 | Male | Intestinal-type |
| 24 | 38 | Female | Intestinal-type |
| 25 | 72 | Female | Intestinal-type |
| 26 | 71 | Male | Intestinal-type |
| 27 | 72 | Male | Intestinal-type |
| 28 | 55 | Male | Intestinal-type |
| 29 | 39 | Female | Diffuse-type |
| 30 | 51 | Female | Diffuse-type |
| 31 | 50 | Female | Diffuse-type |
| 32 | 70 | Male | Diffuse-type |
| 33 | 36 | Female | Diffuse-type |
| 34 | 64 | Male | Diffuse-type |
| 35 | 68 | Male | Diffuse-type |
| 36 | 62 | Female | Diffuse-type |
| 37 | 54 | Female | Diffuse-type |
| 38 | 75 | Female | Diffuse-type |
| 39 | 52 | Male | Diffuse-type |
| 40 | 43 | Male | Diffuse-type |
| 41 | 40 | Male | Diffuse-type |
| 42 | 36 | Male | Diffuse-type |
| 43 | 56 | Female | Diffuse-type |
| 44 | 72 | Female | Diffuse-type |
| 45 | 75 | Female | Diffuse-type |
| 46 | 46 | Female | Diffuse-type |
| 47 | 63 | Female | Diffuse-type |
| 48 | 57 | Male | Mixed-type |
| 49 | 53 | Female | Mixed-type |
| 50 | 49 | Female | Mixed-type |
| 51 | 62 | Male | Mixed-type |
| 52 | 33 | Male | Mixed-type |
| 53 | 64 | Male | Mixed-type |
| 54 | 61 | Female | Mixed-type |
| 55 | 51 | Male | Mixed-type |

Table S5. The comparison against relevant existing EV-miRNA detection methods.

| Method | Technique | EV isolation requirement | Reaction time | Detection range (Particles/µL) | LOD (Particles/µL) | Multiplex detection capability | REF |
| --- | --- | --- | --- | --- | --- | --- | --- |
| This work | Dual-mode | Yes | 30 min | 10^3^-10^9^ | 954.99 | Yes | - |
| CLiAN | nanoflow cytometry | Yes | 2 h | 10^2^-10^6^ | 85 | No | [1] |
| GDEVA | nanoflow cytometry | Yes | 5.5 h | 10^1^-10^5^ | 5 | Yes | [2] |
| CLICK | nanoflow cytometry | No | 2 h | 10-10^8^ | 10 | No | [3] |
| NgCHA | Fluorescence | Yes | 3 h | 10^6^-10^7^ | 2×10^6^ | Yes | [4] |
| EV-CLUE | Fluorescence | No | 1 h | 10^1^-10^6^ | 16 | Yes | [5] |
| siEVPPRA | Fluorescence | Yes | 2 h | 10^3^-10^8^ | 10^3^ | Yes | [6] |
| LPHN–CHDC | Fluorescence | No | 2 h | 10^2^-10^5^ | 60 | No | [7] |
| Zn-TCPP/UiO-66-NH₂ ECL biosensor | Electrochemical | Ye | 1.5h | 1×10⁴-3.16×10⁶ | 9.08×10³ | No | [8] |
| Histostar@COFs | Electrochemical | Yes | 4 h | 10³-10⁸ | 318 | No | [9] |
| Plasma Exosome BACE-1 | Electrochemical | Yes | 2 h | 2.03×10⁴-1.45×10⁷ | 1.51 × 10^4^ | No | [10] |
| GLYPH | SERS | Yes | 1 h | 10^2^-10^6^ | 10^2^ | Yes | [11] |
| S-MMEV | SERS | Yes | 1 h | 10^1^-10^10^ | 0.1 | Yes | [12] |
| MEDP @ H-SERS | SERS | No | 13 h | 10^3^-10^7^ | 10^3^ | Yes | [13] |
| sandwich SERS | SERS | Yes | 2 h | 10⁵-10^6^ | 1.5 × 10^⁵^ | Yes | [14] |
| Nanozyme-Based Lateral Flow Immunoassay | Colorimetric | Yes, | 45 min | 10^7^-10^9^ | 2.49 × 10^7^ | No | [15] |
| DEVM | Colorimetric | Yes | 45 min | 10^3^-10^7^ | 2.4× 10^3^ | Yes | [16] |
| Optofluidics-enhanced color-changing optical metasurface colorimetric assay | Colorimetric | No | 2 min | 10^4^-10^7^ | 10^4^ | No | [17] |

Table S6: All DNA and RNA sequences used in this study are shown in the table below (5’ to 3’ direction).

| Name | Sequence |
| --- | --- |
| 1246 | ATGGATTTTTGGAGCAGG |
| H1 | CTAGCTGTACCCTGCTCCAAAAAT(FAM)CCATGATGTACTAGG(BHQ1)ATGGATTTTTGG |
| H2 | CGATCGATCGATCCATCCTAGTACATCATGGATTTTTGGGATGTACTAGG |
| H3 | TGACTGCTGGCCTAGTACAT(FAM)CCCAAAAAGCAGGATGGATTTTT(BHQ1)GGCCTGCTTTTTGG |
| H4 | TCGAGCAGGCCAAAAATCCATAGCAGGTTTTGGATGGATTTTTGGAGCAGG |
| Capture-1246 | SH-AAAAAAAAAGGACGAAAAACCCTACATGATCCTACCTAAAAACCTCGTCC |
| 183-5p | TATGGCACTGGTACAATTCACT |
| H1 | CTAGCTGTACTATGGCACTGGTACAATTCACTGATGTACTAGGAGTGAATTGTACCA |
| H2: | CGATCGATCGATTCACTCCTAGTACATCAGTGAATTGTACCAGATGTACTAGG |
| H3 | TGACTGCTGGCCTAGTACATCTGGTACAGTGCCATAAGTGAATTGTACCATATGGCACTGTACCA |
| H4 | TCGAGCAGGCGTGCCATATGGTACAATTCACTTATGGCACTGTACCAAGTGAATTGTACCAGTGCCATA |
| Capture-183 | SH-AAAAAAAATACCGTGACATGGTCTACATGATCCTCACTTAACATGGTCACGGTAT |
| 142-5p | CATAAAGTAGAAAGCACTACT |
| H1 | CTAGCTGTACCATAAAGTAGAAAGCACTACTGATGTACTAGGAGTAGTGCTTTCTA |
| H2 | CGATCGATCGCACTACTCCTAGTACATCAGTAGTGCTTTCTAGATGTACTAGG |
| H3 | TGACTGCTGGCCTAGTACATCTAGAAAGCTTTATGAGTAGTGCTTTCTACATAAAGCTTTCTA |
| H4 | TCGAGCAGGCCTTTATGTAGAAAGCACTACTCTTTATGCTTTCTAAGTAGTGCTTTCTACTTTATG |
| Capture-142 | SH-AAAAAAGTATTTCGAAAGATCTACATGATCCTCATCACGAAAGATGAAATAC |
| Nanowire-1-20nt | GCTACGATCGACATGCATCATCGATGCACACGCTAGCTAGCTAGCTAGCTC |
| Nanowire-2-20nt | TTGTGACTGACGACCGATCGATCGATCGACTAGCTAGCTACGATCGATCGA |
| Nanowire-1-15nt | GCTACGATCGACATGCATCATCGATGCACAGCTAGCTAGCTAGCT |
| Nanowire-2-15nt | TTGTGACTGACGACCGATCGATCGATCGACAGCTACGATCGATCG |
| Nanowire-1-10nt | GCTACGATCGACATGCATCATCGATGCTAGCTAGCTAGCT |
| Nanowire-2-10nt | TTGTGACTGACGACCGATCGATCGAAGCTACGATCGATCG |
| Nanowire-1-5nt | GCTACGATCGACATGCATCAGCTAGCTAGCTAGCT |
| Nanowire-2-5nt | TTGTGACTGACGACCGATCGAGCTACGATCGATCG |
| LCHA- Nanowire1 | GCTACGATCGACATGCATCATCGATGCTAGCTAGCTAGCT |
| LCHA- Nanowire2 | TTGTGACTGACGACGATCGATCGAAGCTACGATCGATCG |
| LCHA-H1 | CTAGCTGTACCCTGCTCCAAAAAT(FAM)CCATGATGTACTAGG(BHQ1)ATGGATTTTTGG |
| LCHA-H2 | CGATCGATCGATCCATCCTAGTACATCATGGATTTTTGGGATGTACTAGG |
| LCHA-H3 | TGACTGCTGCCTAGTACAT(FAM)CCCAAAAAGCAGGATGGATTTTT(BHQ1)GGCCTGCTTTTTGG |
| LCHA-H4 | TCGAGCAGGCCAAAAATCCATAGCAGGTTTTGGATGGATTTTTGGCCCCC |

Table S7: The 1 segment and 4segment DNA strand for NUPACK analysis.

| 1 segment | | 4 segment | |
| --- | --- | --- | --- |
| 18 nt | CCTGCTGGATGTACTAGGCCAAAAAT | 18 nt | ATTTTTGCCTGCTGGATGTACTAGG |
| 15 nt | CCTGCTGGATGTACTCCAAAAAT | 15 nt | ATTTTTGGCTGGATGTACTAGG |
| 12 nt | CCTGCTGGATGTCCAAAAAT | 12 nt | ATTTTTGGGATGTACTAGG |
| 9 nt | CCTGCTGGACCAAAAAT | 9 nt | ATTTTTGTGTACTAGG |
| 6 nt | CCTGCTCCAAAAAT | 6 nt | ATTTTTGACTAGG |
| 3 nt | CCTCCAAAAAT | 3 nt | ATTTTTGAGG |

**References**

[1]	Y. Wu, S. Luo, J. Gu, W. Xu, M. Yang, J. Zhou, X. Ma, C. Qiu, M. Xu, G. Huang, L. Zhu, X. Cheng, H. Shen, Q. Cai, F. Wang, *J Nanobiotechnology* **2026**, https://doi.org/10.1186/s12951-026-04780-w.

[2]	J. Park, M. Feng, J. Yang, H. Shen, Z. Qin, W. Guo, D. A. Issadore, *ACS Nano* **2025**, *19* (18), 17884, https://doi.org/10.1021/acsnano.5c04207.

[3]	Y. Fan, W. Pan, Q. Xiu, Y. Qiao, P. Zhang, T. Liao, J. Feng, C. Nie, S. Chu, N. Li, X. Wu, L. Zheng, Z. Cai, B. Li, *Biosens Bioelectron* **2025**, *291*, 118032, https://doi.org/10.1016/j.bios.2025.118032.

[4]	Y. Zhang, Y. Wu, S. Luo, C. Yang, G. Zhong, G. Huang, X. Zhang, B. Li, C. Liu, L. Li, X. Yan, L. Zheng, B. Situ, *ACS Sens* **2022**, *7* (4), 1075, https://doi.org/10.1021/acssensors.1c02717.

[5]	P. Zhang, X. Wu, G. Gardashova, Y. Yang, Y. Zhang, L. Xu, Y. Zeng, *Sci Transl Med* **2020**, *12* (547), https://doi.org/10.1126/scitranslmed.aaz2878.

[6]	J. Zhang, X. Y. Rima, X. Wang, L. T. H. Nguyen, K. Huntoon, Y. Ma, P. L. Palacio, K. T. Nguyen, K. Albert, M. D. Duong‐Thi, N. Walters, K. J. Kwak, M. J. Yoon, H. Li, J. Doon‐Ralls, C. L. Hisey, D. Lee, Y. Wang, J. Ha, K. Scherler, S. Fallen, I. Lee, A. F. Palmer, W. Jiang, S. M. Magaña, K. Wang, B. Y. S. Kim, L. J. Lee, E. Reátegui, *Journal of Extracellular Vesicles* **2023**, *12* (11), https://doi.org/10.1002/jev2.12369.

[7]	J. Hu, Y. Sheng, K. J. Kwak, J. Shi, B. Yu, L. J. Lee, *Nat Commun* **2017**, *8* (1), 1683, https://doi.org/10.1038/s41467-017-01942-1.

[8]	Y. Wang, J. Shu, A. Lyu, M. Wang, C. Hu, H. Cui, *ACS Applied Nano Materials* **2023**, *6* (6), 4214, https://doi.org/10.1021/acsanm.2c05273.

[9]	Y. Lin, B. Nie, X. Qu, M. Wang, J. Yang, G. Li, *Biosensors (Basel)* **2022**, *12* (9), https://doi.org/10.3390/bios12090704.

[10]	R. Rossi, A. Cano, A. Pallares-Rusinol, A. Ruiz, M. Marti, M. I. Pividori, *Biosens Bioelectron* **2026**, *292*, 118061, https://doi.org/10.1016/j.bios.2025.118061.

[11]	Q. Zhou, X. Niu, Z. Zhang, K. O'Byrne, A. Kulasinghe, D. Fielding, A. Moller, A. Wuethrich, R. J. Lobb, M. Trau, *Adv Sci (Weinh)* **2024**, *11* (33), e2401818, https://doi.org/10.1002/advs.202401818.

[12]	X. Chen, J. Tang, Y. Zhao, R. Wang, S. Sang, F. Yu, Y. Xing, *Biosens Bioelectron* **2024**, *267*, 116724, https://doi.org/10.1016/j.bios.2024.116724.

[13]	J. Li, Y. Li, S. Chen, W. Duan, X. Kong, Y. Wang, L. Zhou, P. Li, C. Zhang, L. Du, C. Wang, *Small Methods* **2022**, *6* (6), e2200154, https://doi.org/10.1002/smtd.202200154.

[14]	L. Ngo, W. Zhang, S. S. T. Hnit, Y. Wang, *Analyst* **2023**, *148* (13), 3074, https://doi.org/10.1039/d3an00398a.

[15]	B. Wang, A. Moyano, J. M. Duque, L. Sanchez, G. Garcia-Santos, L. J. G. Florez, E. Serrano-Pertierra, M. D. C. Blanco-Lopez, *Biosensors (Basel)* **2022**, *12* (7), https://doi.org/10.3390/bios12070490.

[16]	Y. Chen, S. Zhang, J. Lu, D. Li, H. Wu, L. Zhang, X. Li, X. Gao, Y. Xu, Z. Zeng, L. Zeng, X. Ding, X. Li, S. Ding, *Small* **2023**, e2208142, https://doi.org/10.1002/smll.202208142.

[17]	C. Hong, I. Hong, S. Yang, J. C. Ndukaife, *Opt Express* **2024**, *32* (4), 4769, https://doi.org/10.1364/OE.506686.
